# Glutathione depletion under hypoxia via a birnessite-type manganese oxide nanozyme inducing immunogenic ferroptosis for magnetic resonance imaging guided cancer therapy

**DOI:** 10.7150/thno.129808

**Published:** 2026-05-11

**Authors:** Huilin Sun, Bo Li, Lin Qiu, Yufeng Zhao, Xin Wang, Chenxian Zhu, Haonan Zhang, Xiang Wang, Yang Bai, Jianhao Wang

**Affiliations:** 1 School of Pharmacy & Affiliated Hospital of Changzhou University (Changzhou West Taihu Hospital), Changzhou University, Changzhou 213164, P. R. China.; 2 Department of Radiology, The Third Affiliated Hospital of Nanjing Medical University, Changzhou 213164, P. R. China.; 3 School of Medical and Health Engineering, Changzhou University, Changzhou 213164, P. R. China.; 4 Department of Gynecology, Changzhou Maternal and Child Health Care Hospital, Changzhou 213000, P. R. China

**Keywords:** nanozyme, manganese oxide, birnessite, glutathione depletion, ferroptosis, immunogenic cell death, magnetic resonance imaging

## Abstract

Glutathione (GSH) depletion can inhibit glutathione peroxidase 4 (GPX4) activity, leading to the accumulation of lipid peroxides and the induction of ferroptosis. However, achieving efficient GSH depletion under the hypoxic conditions typical of tumor microenvironments using non-precious metal-based materials remains a significant challenge for effective cancer therapy.

**Methods:**

We synthesized a two-dimensional birnessite-type nanosheet-like manganese oxide nanozyme (CMO). The multienzymatic activity and GSH consumption capacity of CMO were evaluated. Density functional theory calculations and material characterizations were utilized to investigate the mechanism of GSH oxidation under varying oxygen levels. *In vitro* and *in vivo* experiments using 4T1 tumor models were conducted to assess the nanozyme’s ability to induce cytotoxicity, corresponding mechanism, and function as a magnetic resonance imaging contrast agent.

**Results:**

CMO demonstrated ultrafast GSH depletion, consuming GSH within 1 minute at a concentration of 20 μg/mL. Importantly, this rapid depletion capacity was not suppressed under hypoxic conditions. Theoretical and experimental analyses revealed that GSH oxidation was driven by the reduction of Mn and the sacrificing of lattice oxygen, which generated oxygen vacancies and a rougher material surface. *In vitro* and *in vivo* studies confirmed that CMO induced ferroptosis and immunogenic cell death in 4T1 cells. This process promoted dendritic cell maturation and T-cell infiltration, successfully eliciting a robust systemic antitumor immune response. Furthermore, CMO exhibited significant magnetic resonance imaging capabilities, validating its theranostic potential.

**Conclusions:**

The study presents a non-precious metal-based birnessite-type manganese oxide nanozyme capable of ultrafast GSH consumption under hypoxia. This platform effectively activates immunogenic ferroptosis and offers strong potential for magnetic resonance imaging-guided cancer immunotherapy.

## Introduction

Despite significant advancements in cancer treatment in recent decades, it continues to be a leading cause of mortality worldwide. Current standard interventions including chemotherapy, radiation therapy, and surgical procedures frequently face challenges such as adverse systemic effects and tumor resistance. These limitations highlight the critical demand for novel treatment approaches with improved efficacy and reduced toxicity. Ferroptosis, an iron-dependent form of regulated cell death driven by excessive lipid peroxidation, has attracted extensive attention in the development and therapeutic responses of tumors 1-3. Targeting ferroptosis, which is regulated by various metabolic and immune elements, might become a novel and efficient strategy for antitumor therapy 4-6. The antioxidant enzyme GPX4 could reduce phospholipid hydroperoxide to hydroxyphospholipid, as a major repressor of ferroptosis 7-8. Glutathione (GSH) depletion or deactivation can restrain the GPX4 activity by prohibiting its reactivation cycle and disturb the lipid peroxide-decreasing reaction, leading to the accumulation of LPO in tumor cells to activate ferroptosis 2,9. For example, erastin treatment induces ferroptosis by inhibiting the cystine/glutamate antiporter (system x_c_^-^), leading to the downregulation of cysteine, a precursor of GSH, and thereby reducing GSH synthesis. This, in turn, compromises GPX4 activity due to the lack of its substrate and activates ferroptosis 10. Notably, ferroptosis is characterized by the release of damage-associated molecular patterns (DAMPs), which can promote immunogenic cell death (ICD). The ferroptosis-induced ICD cascade is crucial for activating antitumor immune responses, making it a promising approach to amplify therapeutic efficacy.

Inspired by the development of nanotechnology, GSH-depleting reagents have been successfully established into anticancer nanomedicines 11-13. Integration of the catalytic activities of natural enzymes and distinct properties of nanomaterials renders nanozymes the potential for reactive oxygen species (ROS)-involved tumor therapy 14-16. The construction of multifunctional nanozymes is considered as an ideal strategy to induce multiple intratumoral reactions for selective and efficient cancer therapy 17-19. Transition metal based nanozymes have attracted intense interests in GSH depletion due to their adjustable structures and variable valences that enable them to catalyze or interact with GSH 13,20-23. However, because O_2_ acts as oxidant for the production of oxidized glutathione GSSG, the hypoxic tumor microenvironment (TME) poses major challenges for catalytic GSH consumption. Therefore, approaching high GSH depletion efficiency especially under hypoxia is highly demanded and challenging for cancer therapy.

Manganese (Mn), an essential metal element for human body, serves as the catalytic center metal of natural enzymes such as manganese superoxide dismutase (MnSOD) and catalase (CAT). Mn also garnered significant attention in the nanozyme field as a chemodynamic therapy (CDT) agent 24. Mn based compound such as MnO_2_ consume the GSH *via* undergoing a redox reaction to yield Mn^2+^ and GSSG, and simultaneously exhibit magnetic resonance imaging (MRI) capacity 25. Yang and coworkers developed a nanomedicine DFMC (DMSN/ Fe_3_O_4_-Mn@CB-839) based on dendritic mesoporous silica nano-particles (DMSNs) with integration of Fe_3_O_4_ nanoparticles, Mn^2+^ ions, and glutaminase inhibitor Telaglenastat (CB-839). The GSH depletion induced by DFMC could consume the GSH, block GSH synthesis, and weaken the GSH-related antioxidant defense system, thereby improving the ROS-mediated tumor catalytic therapy 26. Qu et al. have constructed a nanomedicine TDMH (TP/2-DG @HMnO_2_@HA) with triptolide (TP) and 2-deoxy-D-glucose (2-DG) loaded into hollow mesoporous MnO_2_ (HMnO_2_), and then hyaluronic acid (HA) modified on the surface. HMnO_2_ can consume intracellular GSH, and TP/2-DG can inhibit GSH synthesis, thus achieving MRI-guided tumor-specific CDT 27. Shi et al. fabricated a well-dispersed MnOOH nanocatalyst to promote GSH depletion and H_2_O_2_ decomposition to produce abundant ROS and generate an effective superadditive catalytic therapeutic efficacy 22. The GSH interaction activity of the above-mentioned composite nanomedicine relies significantly on the development of core Mn based materials. The GSH-reactive Mn based materials in cancer therapy still face the challenge of efficient GSH depletion with high reaction rate and low reactant concentration.

Birnessite, widely found in natural soils and sediments, represents a class of two-dimensional layered manganese oxides with an interlayer spacing of approximately 0.7 nm. Its layers consist of edge- or corner-sharing manganese-oxygen octahedra (MnO_6_), with interlayer spaces occupied by other metal cations or water molecules 28. The existing one vacancy per every six MnO_6_ octahedra results in a negatively charged octahedral layer that maintains structural stability through electrostatic interactions with intercalated cations 29,30. This unique layered architecture endows Birnessite-type (Bir-type) manganese oxides with remarkable physical and chemical properties, including magnetism, ion exchange capability, catalytic activity, and selective adsorption which inspired us to explore its potential in CDT of cancer 31, 32.

Herein, we report a Bir-type manganese oxides CuMnO_2_ shows a 2D nanosheet-like morphology and exhibits ultrafast GSH consumption capacity and multienzymatic activity. It could deplete the GSH with its concentration of 20 μg mL^-1^ within 1 min, which made it comparable to the most efficient GSH depletion reagent. Moreover, the GSH depletion would not be suppressed under hypoxia, which is a characteristic of TME. On the contrary, CMO treated cells under hypoxia exhibited improved GSSG production and enhanced cytotoxicity. The XPS analysis and density functional theory (DFT) calculation revealed that the sacrificing the lattice oxygen and reduction of Mn contributed to the GSH oxidation, which further resulted in oxygen vacancies and rougher surface. The *in vitro* study revealed the enhanced cytotoxicity under hypoxia (2.01 μg/mL) compared to that under normoxia (47.77 μg/mL), GPX4 downregulation, ROS boosting, and mitochondrial dysfunction to induce LPO accumulation and ferroptosis. ICD has also been confirmed *via* high mobility group protein B1 (HMGB1) release, calreticulin (CRT) exposure, and adenosine triphosphate (ATP) secretion. Together with its MR imaging capacity, CMO has also been confirmed with efficient *in vivo* metabolism, therapeutic effects, and biosafety. The study could make a significant contribution to the development of the GSH depletion reagents based on 2D Bir-type nanozyme for the ultrafast GSH consumption under hypoxia.

## Materials and Methods

### Synthesis of CMO

Cu(NO_3_)_2_•3H_2_O (0.0724 g, 0.299 mmol) and MnCl_2_•4H_2_O (0.0592 g, 0.299 mmol) were dissolved into deionized water (20 mL) and stirred for 30 min. The pH of the mixed solution was adjusted to 13 with the prepared NaOH solution (1 M). Subsequently, the mixture was transferred to a 50 mL stainless steel autoclave lined with polytetrafluoroethylene (PTFE) and heated to 80 ^o^C in an oven for 2 h. Then the autoclave was taken out and aged at 25 ^o^C for 4 h. After washing with deionized water and ethanol for three times, the obtained dark brown precipitate was collected and dried.

### POD-like Activity and Kinetic Assay

In the presence of H_2_O_2_, the peroxidase-like (POD-like) activity of CMO was studied with TMB as the substrate. Different concentrations of CMO (0, 1, 2, 5, 10 μg/mL) were mixed with H_2_O_2_ (100 mM) and TMB (10 mM) in a PBS solution at pH 4. The UV-vis absorbance of the mixture at 652 nm were recorded to investigate the concentration dependence of the POD-like activity of CMO. CMO (10 μg/mL) and H_2_O_2_ (100 mM) were mixed in PBS buffers (with pH of 4, 5, 6, 7) to determine the pH dependence of the POD-like activity.

In enzyme kinetics, the Michaelis constant (K_m_) is the substrate concentration at which the reaction rate is half of its maximum value (V_max_), and it indicates the affinity of a nanozyme for its substrate. The parameter V_max_ represents the maximum reaction rate achieved under saturating substrate conditions. The kinetic constants Km and Vmax were determined by fitting the initial reaction rates (V) against substrate concentration to the Michaelis-Menten equation. The kinetic constants K_m_ and Ѵ_max_ were calculated as:



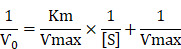



### Glutathione depletion capacity of CMO

The activity of GPX-like was determined by using DTNB as a probe. DTNB reacts with the sulfhydryl (SH) group of glutathione (GSH) to yield a yellow product which absorbs maximally at 412 nm. Since GSH is easy to be oxidized, the experiments were conducted in the dark. In detail, GSH solution (200 μM) was reacted with different concentrations of CMO (0, 1, and 10 μg/mL) in PBS (pH = 4). Subsequently, the CMO was removed by a 220 nm filter membrane. DTNB (100 μM) solutions was added into the supernatant for complete reaction. Then, the absorbance at 412 nm of the mixture was recorded. In order to determine the effect of atmospheric oxygen, the reaction was carried out in air, O_2_ and N_2_ atmosphere respectively.

To evaluate the selectivity of CMO for GSH, the absorbance of DTNB was measured both with and without the introduction of potential interfering molecules. To simulate the intracellular environment, the system was incubated with a mixture of potential interferents (0.17 mM histidine, 0.26 mM arginine, 0.48 mM lysine, 2 mM glucose, 1.6 mM ascorbic acid) and CMO (20 μg/mL) for 20 minutes, after which the GSH content was measured.

CMO (0.1 mg/mL) were mixed with GSH (1 mM) for 12 h for HRMS and XPS analysis.

To investigate the mechanism of GSH consumption, the release of Mn and Cu ions from CMO was measured both in the presence and absence of GSH. Specifically, CMO (250 µL, 0.1 mg/mL) was dispersed into 2.5 mL PBS solutions (pH = 4, 7.4) with or without GSH (0.2 mM). With different reaction time (0, 6, 24 h), 200 μL solution was collected and filtered through 220 nm filter membrane to remove CMO. The resulting solution was subsequently diluted and analyzed by ICP-MS to quantify the released amounts of Mn and Cu.

Different concentrations of CMO (0, 1 and 10 μg/mL) were reacted with GSH solutions (200 μM, PBS solutions, pH = 4) in the absence or presence of H_2_O_2_ (100 μM). DTNB (100 μM) solutions was used to indicate the unreacted GSH and the absorbance of the mixture at 412 nm was compared.

### Intracellular GSH and GSSG Content

4T1 cells were seeded in 60 mm Petri dishes at a density of 10⁶ cells per dish and allowed to adhere overnight. Subsequently, the cells were treated with different concentrations of CMO (0, 10 μg/mL) for 12 hours under either normoxic or hypoxic conditions. Following the treatment, intracellular levels of GSH and GSSG were quantified using a commercial colorimetric kit, in accordance with the manufacturer's protocol. The absorbance at 412 nm was finally measured with a microplate reader.

### *In vivo* antitumor efficiency

All animal experiments performed in this study received prior approval from the Animal Ethics Committee of Changzhou University (Issue No. 20240308030). The procedures were then carried out in strict accordance with the ARRIVE guidelines 2.0 and the “Guidelines for the Care and Use of Laboratory Animals”. Female BALB/c mice (6-8 weeks old, ~20 g) were purchased from the Changzhou Cavens Biological Technology Co., Ltd. (Changzhou, Jiangsu, China). Mice were maintained with SPF food and water for 1-2 week. The animal room temperature is 20-26 ^o^C, warm humidity 40-70%, 12 h of light and darkness alternate and normal feeding before animal experiments.

The *in vivo* tumor model was established by injecting 2 × 10^6^ 4T1 cells into the right subcutaneous side of the mice. When the tumor volume of 4T1 tumor-bearing mice reached 50-100 mm^3^, the mice were randomly divided into two groups as follows: 1) Saline; 2) CMO, and used for antitumor investigation. The tumor volume (V) was obtained based on equation: tumor volume (V) = tumor length × tumor width^2^/2. For the intravenous injection studies. Mice received intravenous injections of either CMO (4 mg/kg) or saline every two days, for a total of four administrations. Throughout this treatment period, tumor volumes and body weights were recorded at two-day intervals. On the fourteenth day, the mice were sacrificed. And, the tumor tissues and organs were harvested for hematoxylin and eosin (HE) staining. Transferase-mediated dUTP nick-end labeling (TUNEL) apoptosis assay and Ki67 were performed on tumor tissues. GPX4 expression was analyzed by immunohistochemistry using a commercially available kit.

### *In vivo* MR (Magnetic Resonance) imaging

Homozygous BALB/c mice were injected with 2 mg/kg of CMO intravenously *via* the tail for MR imaging. The tumor tissues and organs were harvested at different time after injection for observing distribution of CMO by MR imaging system.

### Statistical Analysis

All experimental data are presented as mean ± standard deviation. The student’s t-test was used to assess statistical significances among groups. Quantification of all dyed pictures was done using ImageJ, and statistical analysis was performed using Origin 2022. Statistical significance was represented by *P < 0.05; **P < 0.01; ***P < 0.001;****P < 0.0001.

## Results and Discussion

### Synthesis and Characterizations of 2D Bir-type Nanozyme CMO

**Scheme 1:**
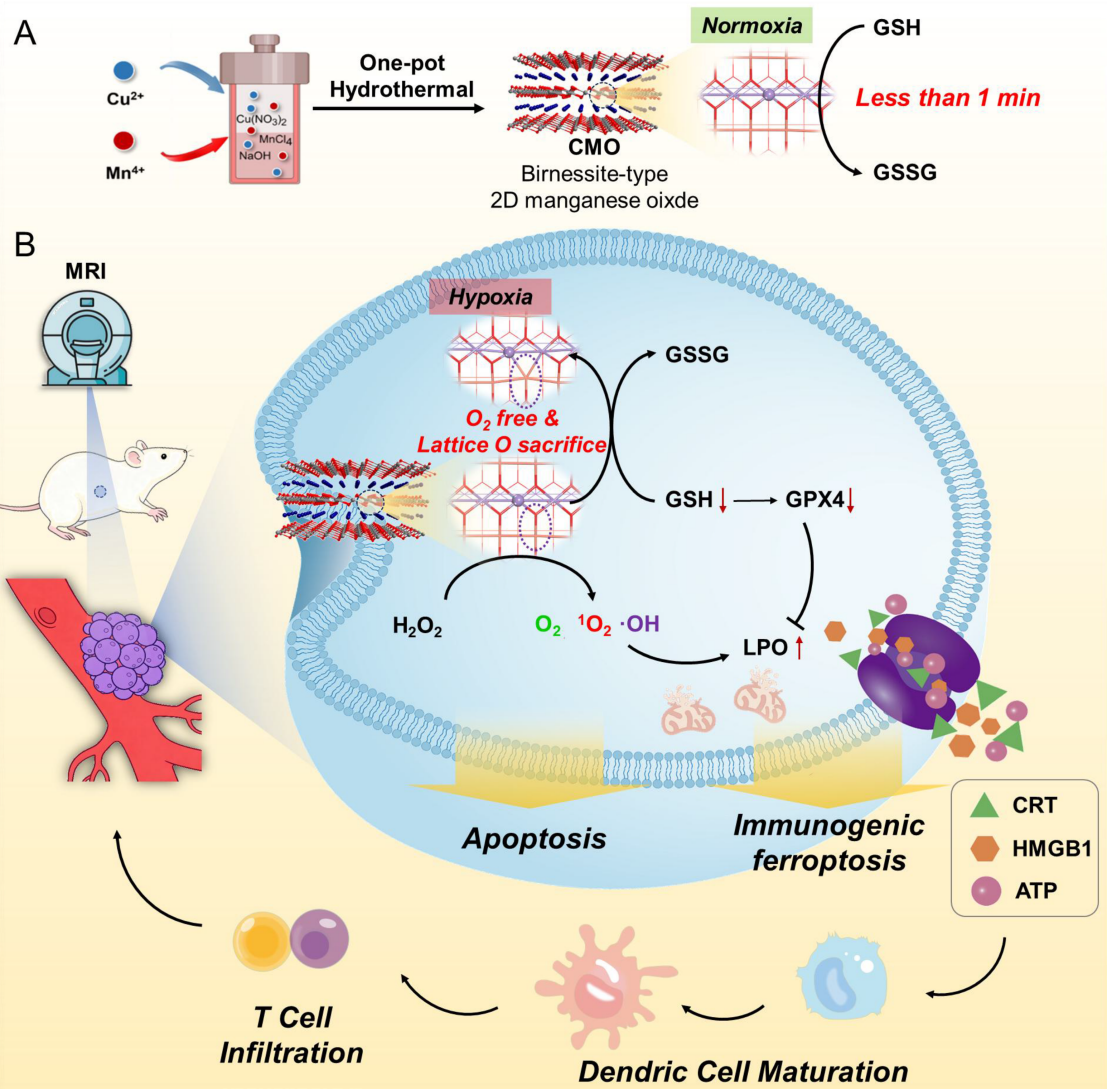
Schematic illustration of (A) the synthesis of Bir-type nanozymes CuMnO_2_ nanosheet (CMO) and (B) its ultrafast GSH depletion, ROS generation and induction ferroptosis and ICD for cancer immunotherapy.

**Figure 1:**
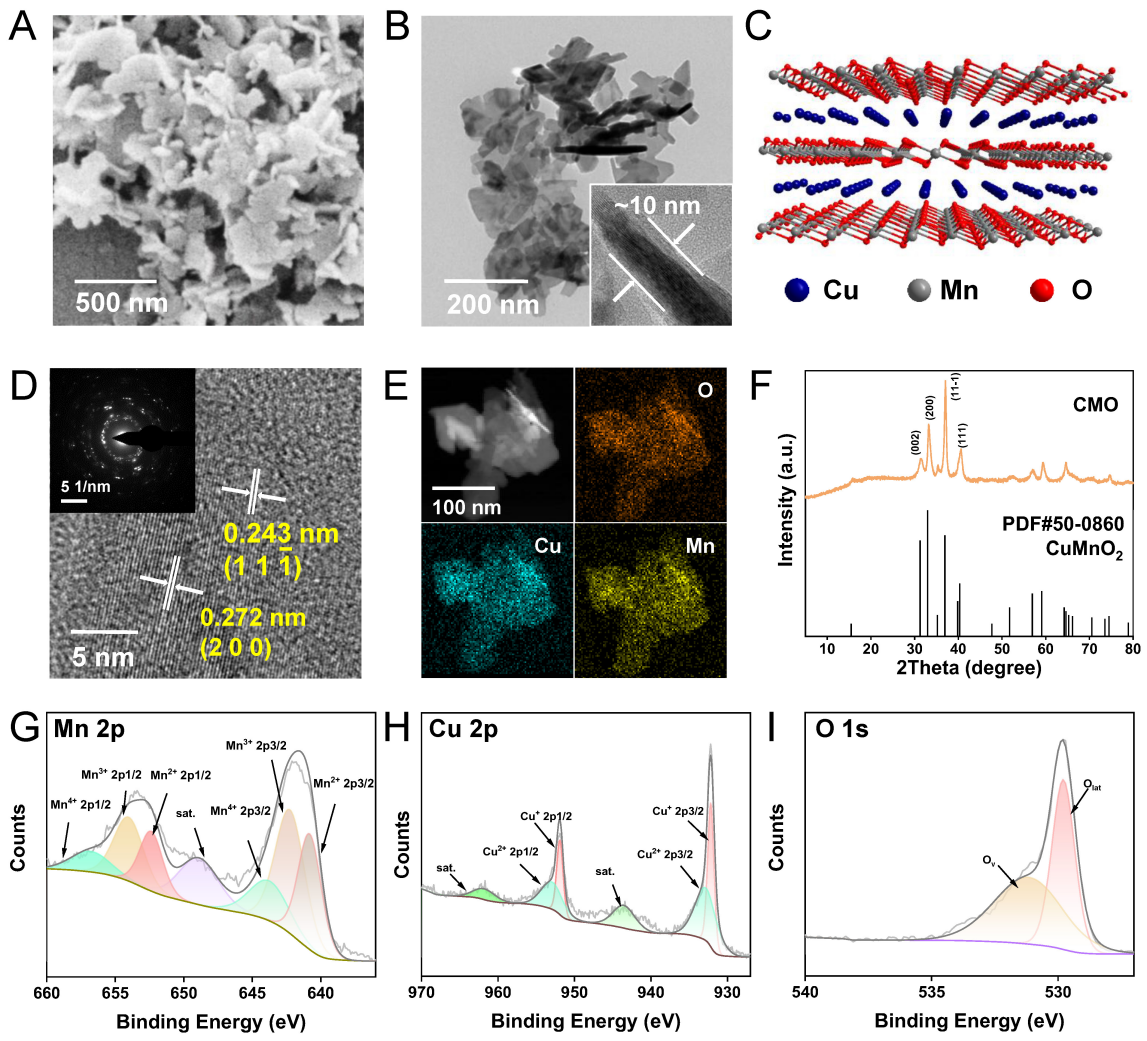
Sample preparation and characterization. (A) SEM image of CMO. Scale bar: 500 nm. (B) TEM image of CMO. Scale bar: 200 nm. Inset: representative side view of the nanosheet. (C) Crystal structure of CuMnO_2_ along b axis. (D) HRTEM image of CMO. Scale bar: 5 nm. Inset: corresponding SAED pattern. (E) HAADF image and elemental mapping of CMO. Scale bar: 100 nm. (F) XRD pattern of CMO. (G-I) High resolution Mn 2p (G), Cu 2p (H), and O 1s (I) XPS spectra of CMO.

The CMO was synthesized *via* a facile one-pot solvothermal approach, utilizing MnCl_2_∙4H_2_O and Cu(NO_3_)_2_∙3H_2_O with NaOH aqueous solution as a structural-directing agent. CMO was then modified with 1,2-distearoyl-sn-glycero-3-phosphoethanolamine-polyethylene glycol (DSPE-PEG) which is a biocompatible, biodegradable, and amphiphilic phospholipid-polymer conjugate widely used in drug delivery (Scheme 1) 33. Scanning electron microscope (SEM) and transmission electron microscope (TEM) images displayed a sheet-like 2D morphology, with an average lateral size of approximately 250 nm and thickness as low as 10 nm (Figure 1A-B). The aspect ratio as large as 25 would contribute to the exposure of the active site. Dynamic light scattering (DLS) particle size distribution analysis showed the averaged particle size was 250 nm, consistent with the SEM and TEM observations (Figure S1A). The stability of CMO was evaluated by monitoring its hydrodynamic size in water, physiological saline, PBS, DMEM, and DMEM supplemented with 10% FBS over 7 days. No significant size variation was observed in these GSH-free environments across all tested media, confirming its structural integrity under non-reactive conditions (Figure S1B). The 2D sheet-like morphology was attributed to the layered structure of the Bir-type Manganese oxides (Figure 1C). It is noteworthy that the introduction of Cu in a specific stoichiometric ratio is a prerequisite for the formation of this ultrathin nanosheet structure. The high-resolution transmission electron microscopy (HRTEM) image of the nanozyme revealed distinct lattice fringes, with interplanar spacings of 0.272 nm and 0.243 nm, which correspond to the (200) and (11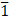) crystal planes of CuMnO₂, respectively. (Figure 1D). The elemental mapping revealed that Cu, Mn, and O were evenly distributed throughout CMO (Figure 1E). X-ray diffraction (XRD) pattern showed that the well-defined and sharp diffraction peaks were in accordant with Bir-type CuMnO_2_ (PDF No. 50-0860, Figure 1F). The main diffraction peaks at 31.2, 32.9, 36.9 and 40.4° were indexed to the (002), (200), (11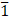), and (111) planes of CuMnO_2_, respectively. The elemental composition and chemical states of the material were investigated by X-ray photoelectron spectroscopy (XPS). (Figure S2). The high-resolution Mn 2p spectra could be resolved into peaks at 640.6, 641.8 and 643.8 eV, which could be attributed to Mn^2+^, Mn^3+^ and Mn^4+^ (2p3/2), respectively (Figure 1G) 34-36. The high-resolution Cu 2p spectra could also be resolved into the peaks located at 930.7 eV (Cu^+^ 2p3/2) and 933.6 eV (Cu^2+^ 2p1/2, Figure 1H). In the deconvoluted high resolution O 1s spectra, the peak located at 529.6 eV corresponds to lattice oxygen out of the metal–oxygen bonding and the one located at 531.2 eV is assigned to the oxygen vacancy (Figure 1I) 37-38. Those results validated the ultra-thin 2D nanosheet morphology of CMO with Bir-type CuMnO_2_ structure 37.

### Ultrafast GSH Depletion and Multienzymatic Activities of CMO

**Figure 2:**
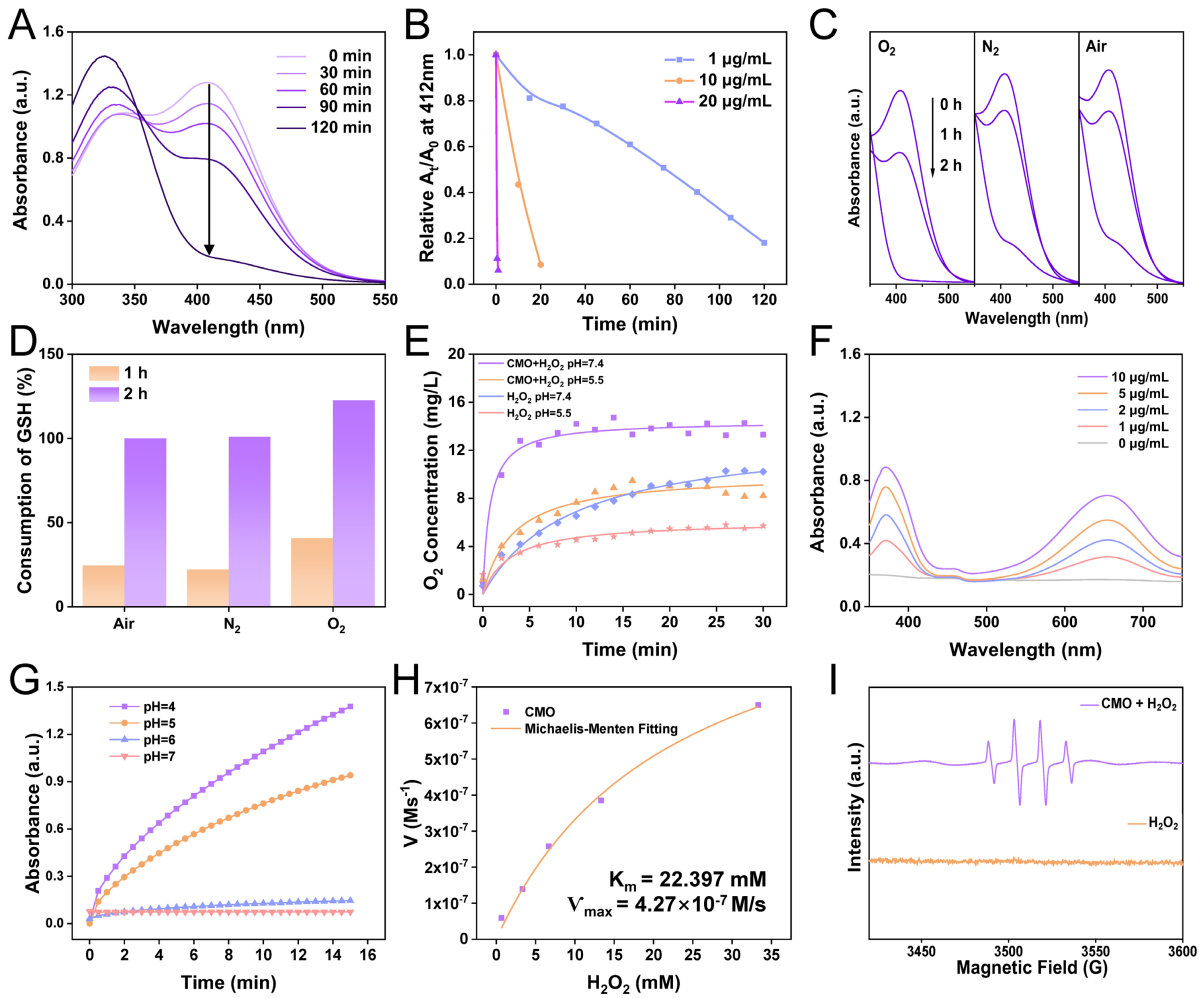
Multienzymatic activities. (A) UV-vis absorption of 0.1 mM DTNB after reaction with the mixture of 0.2 mM GSH and 1 μg/mL CMO with different CMO incubation time. (B) The ratio (A_t_/A_0_) of the absorbance at 412 nm of 0.1 mM DTNB with the mixture of 0.2 mM GSH and CMO with different concentrations. (C) The absorption spectra of 0.1 mM DTNB with the mixture of 0.2 mM GSH and 1 μg/mL CMO with different incubation time under air, N_2_, or O_2_ atmosphere. (D) The ratio [(A_0_-A_t_)/A_0_] of the absorbance at 412 nm of 0.1 mM DTNB with the mixture of 0.2 mM GSH and 1 μg/mL CMO with different incubation time under air, N_2_, O_2_ atmosphere. (E) Dissolved O_2_ of 100 mM H_2_O_2_ with or without 20 μg/mL CMO in PBS solutions with different pH values. (F) UV-vis absorption of 0.6 mM TMB reacted with different concentration CMO and 0.66 mM H_2_O_2_ in PBS soulutions (pH = 4). (G) Time-lapse absorbance at 652 nm of 0.6 mM TMB reacted with CMO (10 μg/mL) and 0.66 mM H_2_O_2_ at different pH conditions. (H) Kinetic assay for the POD-like activity of CMO with H_2_O_2_ as substrate. Inset: calculated K_m_ and Ѵ_max_ values. (I) ESR signals of H_2_O_2_ in the absence (orange) and presence of 20 μg/mL CMO (purple) using DMPO as the trapping agent.

Ferroptosis is highly related to the consumption of GSH. GSH consumption is one of the major strategies for nanozymes to trigger ferroptosis in tumor cells 39. Hence the ability of GSH depletion of CMO was explored *via* the colorimetric reduction from DTNB [5,5'-Dithiobis-(2-nitrobenzoic acid)] to its monomer TNB. The absorbance of TNB was decreased in a dose and time-dependent pattern. Upon the addition of 1 μg/mL CMO, the absorbance of TNB at 412 nm decreased significantly, and GSH was nearly completely depleted within 2 h (Figure 2A). Additionally, the elevated concentrations of CMO led to accelerated GSH consumption. When the CMO concentration attained 20 μg/mL, 1 min was merely required to complete the reaction, which made it comparable to that of state-of-the-art GSH depletion reagents (Figure 2B, Table S1). The reported GSH depletion strategies rely mostly on an oxygen-consumptive catalysis, which could be hindered in the hypoxic tumor microenvironment (TME). The oxygen dependence of the depletion of GSH of CMO was further evaluated in air, nitrogen and oxygen atmosphere respectively. Although the reaction in O_2_ saturated solutions was promoted, the GSH consumption behaviors of CMO in deoxygenated solutions within 1 h and 2 h were comparable to those conducted in air (Figure 2C-D). The results indicated that CMO can also deplete GSH rapidly even in the absence of oxygen, suggesting its potential in the hypoxic TME. Besides, the GSH depletion of CMO was barely interfered with by the presence of the oxidizing H_2_O_2_ in tumor environment; on the contrary, it was slightly enhanced. (Figure S3). Moreover, the oxidation by CMO is selectively toward GSH without interference from other compounds in a physiological environment (Figure S4).

Transition metal oxides are a class of nanozymes which are featured as multi-enzymatic activity. In virtue of the nanozymes, H_2_O_2_ can be decomposed into the highly toxic •OH by POD mimics, or O_2_ by catalase (CAT) mimic, which contribute to the antitumor effects 38,40. To verify the CAT-like activity of CMO, O_2_ concentration of CMO and H_2_O_2_ mixtures was recorded. Dissolved O_2_ was increased to 9.1 and 12.5 mg/L within 15 min in acidic and neutral PBS solutions, respectively, in the presence of CMO (Figure 2E, S5).

To examine the POD-like activity of CMO, a typical colorimetric analysis based on 3,3′,5,5′-tetramethyl-benzidine (TMB) was employed. In the presence of H_2_O_2_, CMO can catalyze the oxidation of TMB to yield blue-colored oxTMB with typical absorbances at 370 and 652 nm. The nanozymes exhibited a 4-fold enhancement of the absorbance at 652 nm, showing a dose-dependently peroxidase-like activity (Figure 2F). The impact of pH on the POD-like activity of CMO was analyzed (Figure 2G and S6). The absorbance of oxTMB at 652 nm in acidic environment at pH 4, 5, or 6 revealed 15-, 13- and 5-fold increases, respectively, compared to the neutral group (pH 7). The result indicated that the POD-like activity of CMO is more active in acidic TME compared to normal physiological condition, while the CAT-like activity is relatively limited. The Michaelis constant (Kₘ) and maximal velocity (V_max_) for CMO in the catalytic oxidation of H_2_O_2_ were determined to be 22.397 mM and 4.27×10⁻⁷ M/s, respectively, consistent with Michaelis-Menten kinetics. (Figure 2H). The generated free radical was further analyzed to be •OH utilizing the electron spin resonance (ESR) spectra, which confirmed the POD activity of CMO (Figure 2I). It has been reported that the toxic singlet oxygen (^1^O_2_) could be generated from O_2_ without photosensitization 41. Using 1, 3-diphenylisobenzofuran (DPBF) as the indicator, the production of ^1^O_2_ was observed when CMO was dissolved in buffer solution, confirmed *via* the triplet ESR signals (Figure S7). Taking advantage of the catalase-like activity, a cascade reaction can be reached upon the addition of H_2_O_2_ in the CMO aqueous solution, showing an enhanced ^1^O_2_ yield.

In order to investigate the influence of antioxidant GSH towards the ROS generation, the CMO-catalyzed ROS generation in the presence or absence of GSH was detected using 2’, 7’-dichlorodihydrofluorescein (DCFH), which could undergo oxidation by active ROS, resulting in fluorescence emission at 525 nm (Figure S8). It revealed that the CMO could catalyze H_2_O_2_ into ROS regardless of the presence of GSH, which could be attributed to the rapid and complete GSH depletion before the catalysis of H_2_O_2_ into ROS. Combined with the GSH depletion promotion by H_2_O_2_, the GSH depletion and ROS production could be realized simultaneously by CMO which would contribute to its application in TME.

### Mechanism of GSH depletion of CMO under normoxia and hypoxia

**Figure 3:**
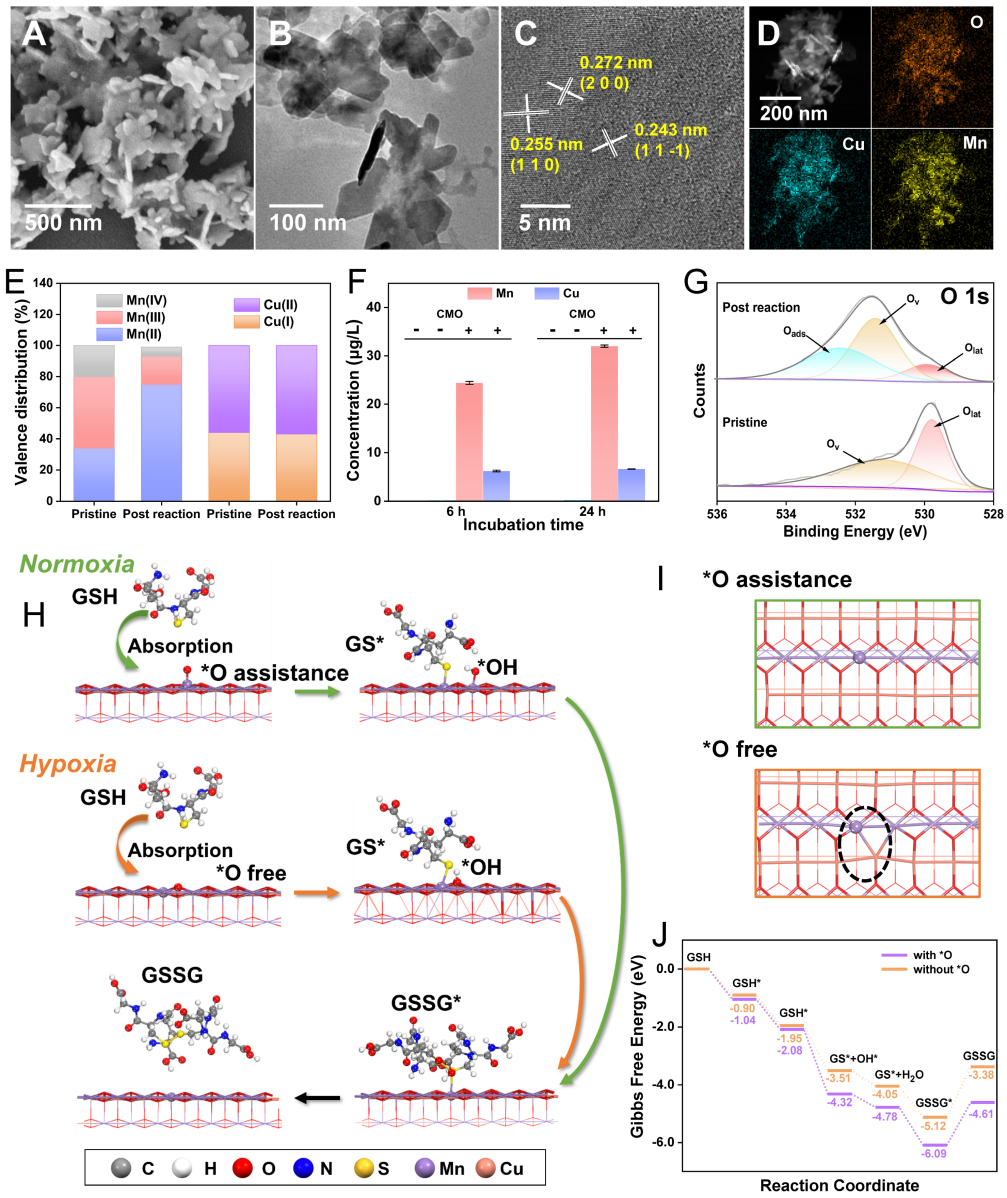
Glutathione depletion mechanism study. (A-C) SEM (A), TEM (B), HRTEM (C) images, and HAADF image/elemental mapping (D) of CMO after reaction with GSH for 6 h. (E) Valence distribution of Mn and Cu before and after the reaction with GSH according to high resolution Mn 2p and Cu 2p spectra. (F) ICP-MS analysis of Mn and Cu ions in the reaction mixture system with 0.2 mM GSH and 100 μg/mL CMO in PBS solutions (pH = 4). (G) High resolution O 1s XPS spectra of CMO before and after reaction with GSH. (H) Proposed molecular mechanism of GSH oxidation on the (200) facet of CMO. The gray, white, red, blue, yellow, purple, and pink balls represent the C, H, O, N, S, Mn, and Cu atoms, respectively. (I) View towards (200) facet of the CMO after reaction with GSH with (up) or without (bottom) *O according to the DFT calculation. Dash circle: catalytic site. (J) Free energy diagram of the GPX-like reaction process with or without the assistance of *O.

Morphological and structural studies were conducted to further investigate the origination of the ultrafast GSH depletion of CMO even under hypoxia circumstance. After the reaction with GSH, the residue of CMO was characterized *via* SEM, TEM, HRTEM, XRD and XPS. The supernatant was also analyzed through inductively coupled plasma mass spectrometry (ICP-MS) and high-resolution mass spectrometry (HRMS). It can be observed that the nanosheet-like morphology (Figure 3A-B) with surficial crystal facets (200) and (11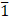) (Figure 3C) and elemental distributions (Figure 3D) remained almost unchanged of the post-reaction residues. Noteworthily, the peaks in the diffraction patterns were slightly reduced of approximately 0.2^o^ compared to the pristine CMO (Figure S9, Table S2). Besides, the binding energies of all existing forms in the high resolution Mn XPS spectra were also shifted to higher binding energy by 0.98 eV, indicating the charge transfer and lattice distortion (Figure S10B). The XPS survey spectra of CuMnO₂ before and after the reaction (Figure S2, S10A, respectively) showed negligible changes in elemental composition. To uncover the structural changes during this process, quantitative XPS analysis was performed (Table S3). The high-resolution Mn 2p spectra revealed that the content of Mn(III)/Mn(IV) decreased from 46.6%/19.3% to 18.4%/6.9%, respectively, while the proportion of Mn(II) surged remarkably from 34.1% to 74.7% after the reaction (Figure 3E). This massive reduction of the metal centers indicated severe charge transfer and structural alteration. In addition, the Mn content in the supernatant after 6 h and 24 h reaction was measured to be 24.3 and 32.0 μg/L, respectively, *via* ICP-MS, which revealed the leakage of Mn out of the lattice with increasing reaction time (Figure 3F). On the contrary, Cu(I) and Cu(II) in the residue *via* XPS analysis showed negligible changes in neither binding energy or relative proportion (Figure S10C). The supernatant also showed less Cu leakage compared to that of Mn (Figure 3F). Dictated by its interlayered position within the Birnessite structure, this phenomenon does not indicate that Cu is inactive. It suggested that interlayer Cu likely functions as a structural stabilizer and electronic mediator, facilitating rapid electron transfer between adsorbed GSH and the reactive Mn center through transient redox cycling. Furthermore, the deconvoluted high-resolution O 1s spectra provided direct quantitative evidence for the emergence of oxygen vacancies (Figure 3G). Compared to the pristine CMO, the post-reaction spectra exhibited a substantial decrease in the relative proportion of the lattice oxygen (O_lat_) peak. Consequently, the calculated ratio of oxygen vacancies to the total structural oxygen, O_v_/(O_v_ + O_lat_), increased from 0.54 to 0.79. Concurrently, an adsorbed oxygen (O_ads_) peak emerged, as the newly generated oxygen vacancies act as highly reactive defect sites that readily capture surrounding oxygen species. HRMS indicated that the main product of GSH reacting with CMO was GSSG (Figure S11). Collectively, the substantial Mn leakage, distinct valence reduction, and O 1s binding energy shifts confirmed that the GSH was depleted via the consumptive reduction of the CMO framework. In this bimetallic catalytic system, the two metals act synergistically. The Mn-O octahedra form the primary exposed reactive facets, the lattice Mn serves as the dominant redox-active center, participating directly and extensively as the oxidant. Meanwhile, the interlayered Cu cations function as an essential electronic mediator, facilitating rapid electron transfer between the adsorbed GSH and the reactive Mn center through transient redox cycling. This synergistic bimetallic contribution dictates the ultrafast GSH oxidation kinetics even under hypoxic conditions.

To uncover the underlying mechanism, the GSH oxidation behaviors at the atomic level of CMO in normoxia and hypoxia were further simulated *via* DFT calculations. Bir-type CuMnO_2_ is a typical 2D layered structure, composed of alternating stacked Mn–O layers with edge-shared MnO_6_ octahedra as basic unit and interlayered Cu cations 30. Due to the highly exposed lateral dimension of the sheet-like morphology, (200) facet of CMO was selected as the active site. In assistance of O_2_, O atom would be absorbed on (200) facet of CMO to support the dissociation of GSH (Figure 1C). The GSH* and OH* that were absorbed can react to produce GS* on (200) facet and generated a water molecule. The GSH* and O* that were absorbed could also undergo a reaction to produce GS* and OH* in the absorbed state. The resulting OH* could proceed to react with other GSH*, or it could alternatively react with a hydrogen atom to produce a water molecule. Ultimately, the generated GS* could form GSSG through coupling interaction (Figure 3H and S12). In a hypoxic TME, the GSH oxidation is believed to be in a similar process without the assistance of O_2_ (Figure 3H and S13). In the O_2_-free path, CMO can provide an O atom for the oxidation by sacrificing its own crystalline structure, producing an oxygen vacancy (Figure 3I, dash circle). The ΔG during the reaction is -4.61 and -3.38 eV under normoxia and hypoxia, respectively (Figure 3J). Even though the energy barrier for the reaction was lower in normoxia, the consecutive consumption of dissolved O_2_ hindered the rapid GSH oxidation in the O_2_-assistance path. The oxygen vacancies and lattice distortion created in hypoxic GSH oxidation resulted in the rougher crystal surface and structural instability, which further contributed to the accelerated GSH consumption and Mn leakage. The proposed mechanism was completely consistent with the experimental analysis of the post-reaction nanozyme.

### *In vitro* Antitumor Effects and Activation of Ferroptosis of CMO

**Figure 4:**
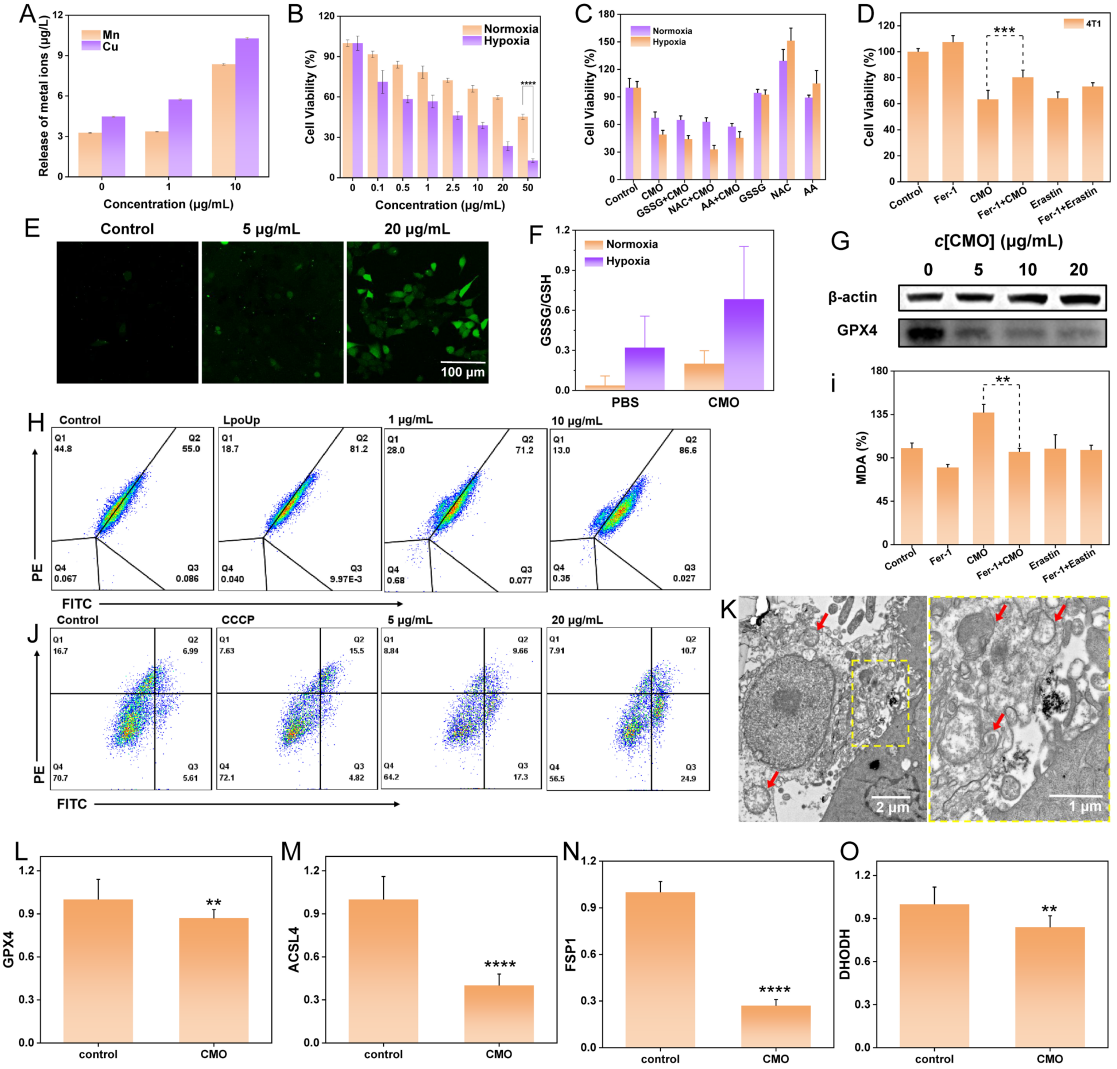
*In vitro* anticancer study. (A) ICP-MS analysis of Mn and Cu ions of CMO incubated 4T1 cells after 24 h. (B) Cytotoxicity of 4T1 cells treated with CMO under normoxia and hypoxia condition. All data are presented as means ± SD (n = 6 independent experiments). Significance between two groups was assessed by Paired Sample t-test. (C) Cytotoxicity of 4T1 cells treated with CMO, the mixture of antioxidants and CMO, antioxidants under normoxia and hypoxia condition. (D) Cell viability of 4T1 cells after different treatments. (E) Confocal images of DCFH-DA stained 4T1 cells after incubating with CMO of different concentrations for 4 h. (F) GSSG/GSH level in 4T1 cells after CMO treatment under normoxia and hypoxia. (G) Western blot results for GPX4 expression levels in 4T1 cells after treatment with CMO. (H) LPO level of 4T1 cells after incubation of different concentrations of CMO for 12 h. (I) The content of MDA of 4T1 cells after different treatments for 12 h. (J) Flow cytometry analysis of JC-1 stained 4T1 cells treated with CMO. (K) TEM images of 4T1 cells after incubating with CMO (left) and corresponding enlarged image of the yellow dash square. Scale bar: 2 μm in left and 1 µm in right. Red arrow: representative mitochondria. (L-O) Relative expression of GPX4(L), ACSL4(M), FSP1(N), and DHODH(O). All data are presented as means ± SD (n = 3 independent experiments for (L-O)). Significance between two groups was assessed by Paired Sample t-test.

Cellular internalization is a fundamental prerequisite for employing nanomaterials *in vivo*. Cu and Mn content of 4T1 cells after an incubation of 10 μg/mL CMO for 24 h were monitored by ICP-MS which exhibited increments over 2.5- and 2.3-fold compared to the intact cells, indicating efficient cellular uptake (Figure 4A). The cytotoxicity of CMO was assessed in 4T1 cells under normoxia and hypoxia *via* methyl thiazolyl tetrazolium (MTT) method. After 50 μg/mL of CMO incubating for 24 h, only 45.3% of the cells were viable. Importantly, the cytotoxicity of CMO in hypoxia was evidently enhanced compared to that in normoxia under 50 μg/mL CMO treatment in which 12.8% of the cells were viable (Figure 4B). IC_50_ was calculated to be 47.77 and 2.01 μg/mL under normoxia and hypoxia, respectively. In addition to 4T1 murine breast cancer cells, the ferroptotic activity of CMO was validated in MCF-7 human breast cancer cells, where concentration-dependent cytotoxicity was observed and reversed by Fer-1 (Figure S14A, S15). Moreover, CMO exhibited negligible cytotoxicity toward NIH/3T3 normal mouse fibroblasts, indicating favorable tumor selectivity (Figure S14B). The CMO-induced cytotoxicity was also observed by employing Calcein-AM/propidium iodide to label live/dead cells (Figure S16). To dissect the mechanism by which CMO exhibits stronger cytotoxicity under hypoxia, we evaluated the effects of exogenous antioxidants using MTT assays, aiming to distinguish whether hypoxia enhances CMO activity or sensitizes cells to CMO. As shown in Figure 4C, co-treatment with N-Acetylcysteine (NAC), Ascorbic Acid (AA), or GSSG under hypoxia failed to rescue CMO-induced cell death, with viability remaining as low as in the CMO-alone group. Given that the antioxidants were bioactive, their inability to reverse the effect suggests that hypoxia primarily enhances the intrinsic toxicity of CMO. This biological outcome highly corroborated the physiochemical investigations (Figure 3). It confirmed that CMO underwent an aggressive structural breakdown under hypoxia via lattice oxygen sacrifice and rapid Mn leakage. Consequently, this breakdown intrinsically amplified its oxidative capacity and cytotoxicity. This enhanced toxicity operated independently of the basal cellular antioxidant defense. To confirm the involvement of ferroptosis, rescue experiments were performed using the ferroptosis inhibitor Ferrostatin-1 (Fer-1). As assessed by MTT assays, CMO-induced cytotoxicity was significantly reversed by Fer-1 (Figure 4D). The remarkably enhanced cytotoxicity of CMO in hypoxia endow it potential for application in hypoxic TME.

Taking advantage of the effective endocytosis and ROS generation ability of CMO, the intracellular ROS level induced by CMO was subsequently evaluated using confocal laser scanning microscope (CLSM) and flow cytometry with 2’,7-dichlorofluorescin (DCFH-DA) staining. The CLSM images exhibited a distinct green fluorescence in the 4T1 cells treated with CMO (Figure 4E). While ferroptosis is a primary mechanism driven by GPX4 deactivation, the prominent Annexin V-FITC/PI positive populations (Figure S17) indicate that CMO also effectively induces concurrent apoptosis. Furthermore, there was a significant dose-dependent elevation in ROS levels in 4T1 cells, which was also identified as 2.6- and 8.0-fold at 1 μg/mL and 10 μg/mL according to the flow cytometry analysis (Figure S18). Cellular redox imbalance, characterized by decreased GSH levels, is also recognized as a critical feature of ferroptosis 4. Owing to the capacity of CMO for rapid oxidation of GSH under both normoxia and hypoxia, the ratio of intracellular GSSG/GSH of the CMO treated 4T1 cells was significantly increased to 5.4 and 2.1-fold under normoxia or hypoxia, respectively, indicating excellent GSH oxidation capacity of CMO in TME (Figure 4F). GSH depletion can deactivate GPX4 in cells and impede lipid repair mechanisms, leading to ferroptosis 42. GPX4 expression in 4T1 cells exhibited an inverse correlation with increasing concentrations of CMO (Figure 4G, S19). Excessive ROS production and down-regulation of GPX4 can lead to the of LPO accumulation, which is a key marker of ferroptosis 43. The LPO assays demonstrated a significant increase, dose-dependent in LPO levels in the 4T1 cells treated with CMO for 12 h (Figure 4H, S20). To further evaluate lipid peroxidation, the levels of malondialdehyde (MDA) were measured. MDA is a stable end product of polyunsaturated fatty acid peroxidation. As shown in Figure 4I, CMO treatment significantly increased MDA levels compared to the control group. Co-treatment with the ferroptosis inhibitor Fer-1 markedly attenuated this increase, indicating that CMO-induced lipid peroxidation is a key event in ferroptosis. Furthermore, the classical ferroptosis inducer Erastin was introduced as a positive control. As shown in Figure 4D and Figure S15, CMO exhibited potent cytotoxicity comparable to Erastin in both 4T1 and MCF-7 cells, and the cell death in both groups was significantly rescued by Fer-1. Similarly, CMO induced a substantial accumulation of MDA that was comparable to the Erastin-treated group (Figure 4I), confirming its robust ferroptosis-inducing capability.

The disturbance of mitochondrial membrane potential (MMP) and mitochondrial morphology were evaluated as essential ferroptosis associated features. Evident MMP depolarization detected by JC-1 staining emerged with increased concentrations of CMO and the resulting improved oxidative stress injury (Figure 4J) 44. Furthermore, the bio-TEM images revealed the swelling morphology of mitochondria with shrunken and decreased cristae in CMO-treated cells (red arrows in Figure 4K, S21). The loss of mitochondrial membrane potential and the subsequent massive ROS generation act as triggers of both apoptosis and ferroptosis. The oxidative stress simultaneously initiates lipid peroxidation-driven ferroptosis and mitochondria-dependent intrinsic apoptosis, establishing a lethal crosstalk that maximizes cytotoxicity.

The expression levels of key ferroptosis-related genes were examined by quantitative real-time PCR. As shown in Figure 4L-O, CMO treatment significantly downregulated the expression of GPX4, ACSL4, FSP1, and DHODH, while GCLC expression was significantly upregulated (Figure S22). The downregulation of GPX4, a central negative regulator of ferroptosis, along with the modulation of FSP1 and DHODH, supports the activation of ferroptotic pathways. Interestingly, ACSL4, which is typically upregulated during ferroptosis to provide polyunsaturated fatty acid substrates for lipid peroxidation, was downregulated in this system. This may reflect a negative feedback mechanism in response to excessive and rapid lipid peroxidation induced by CMO, as cells may attempt to limit further substrate supply. The upregulation of GCLC, the rate-limiting enzyme for glutathione synthesis, is likely a compensatory stress response via the Nrf2 pathway, triggered by the rapid depletion of GSH. All of the results proved the CMO-induced ferroptosis in 4T1 cells.

**Figure 5:**
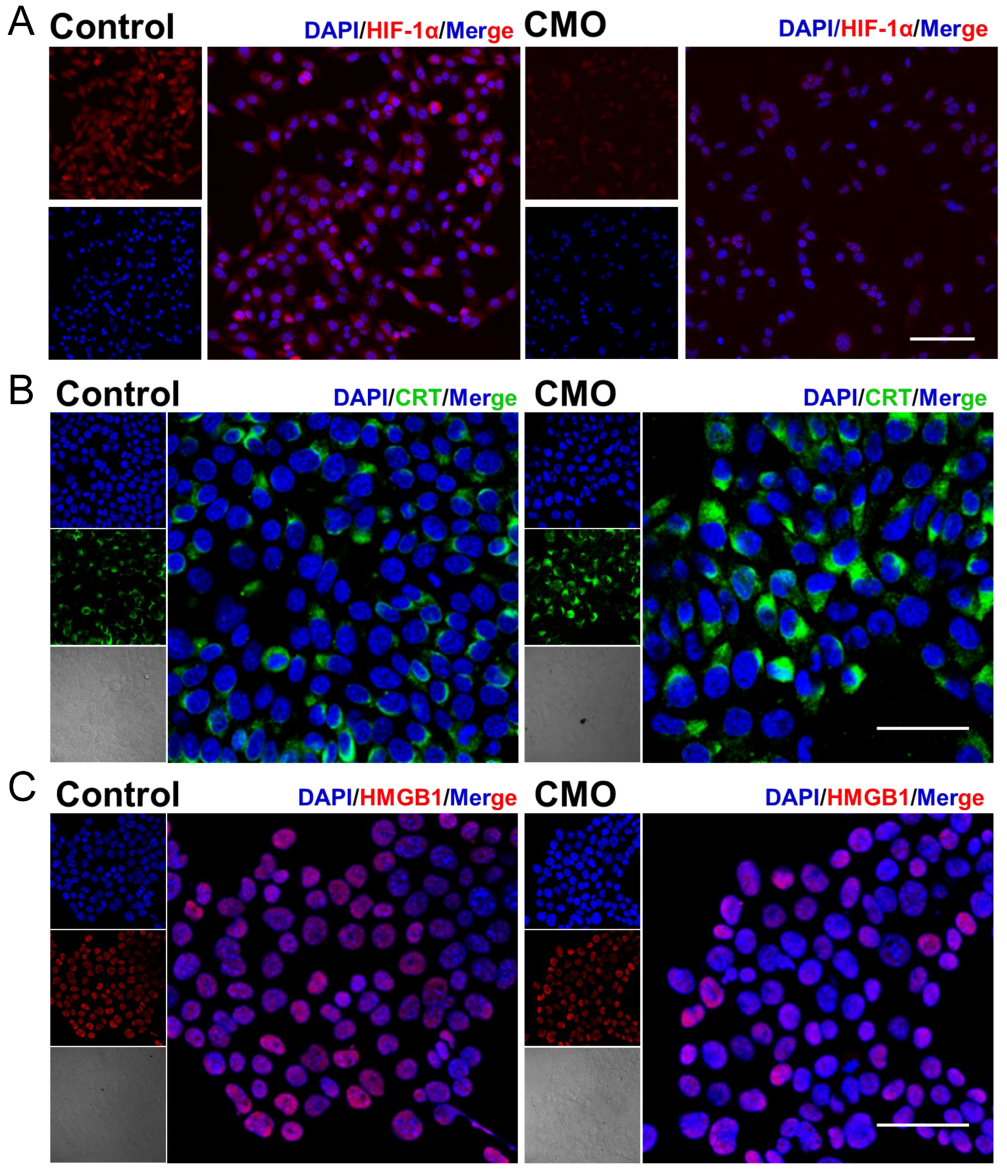
HIF-1α downregulation and immunogenic cell death induction in CMO-treated cells. (A) Immunofluorescence images of HIF-1α expression in 4T1 cells after various treatments. Scale bar =100 μm. (B-C) Confocal imaging of cellular protein expression levels of CRT and HMGB1 in 4T1 cell. Scale bar = 50 μm.

To assess whether HIF-1α signaling is involved in CMO-induced cell death, HIF-1α protein expression has been examined by immunofluorescence staining. HIF-1α fluorescence intensity was markedly decreased in CMO-treated cells compared with control cells, indicating downregulation of HIF-1α protein under this condition (Figure 5A). The result suggested that the HIF-1α signaling pathway is not activated and is unlikely to play a major role in regulating iron metabolism or antioxidant gene expression during CMO-induced cell death. CDT-mediated cell death would cause ICD, which could synergistically promote the cancer therapy. The following hallmarks of ICD were evaluated in 4T1 cells: calreticulin (CRT) surface exposure, high mobility group box-1 (HMGB1) release, and adenosine triphosphate (ATP) secretion. Confocal imaging revealed intense CRT-associated fluorescence (green fluorescence) across the entire cell, excluding the nucleus, following CMO administration which indicated its translocation (Figure 5B). Moreover, a reduction in nuclear HMGB1 levels (red fluorescence, Figure 5C) was observed upon CMO treatment, suggesting its cytoplasmic release. As mitochondria produce ATP by utilizing the proton electrochemical gradient potential across the mitochondrial membrane, tricarboxylic acid cycle and ATP production are highly related to MMP, lipid peroxide accumulation, and ferroptosis 45. The cellular ATP level was decreased by 2.5-/1.4-/1.1-fold after the treatment of 20/10/5 μg/mL CMO, compared to the intact cells, which showed remarkable ATP efflux (Figure S23).

**Figure 6:**
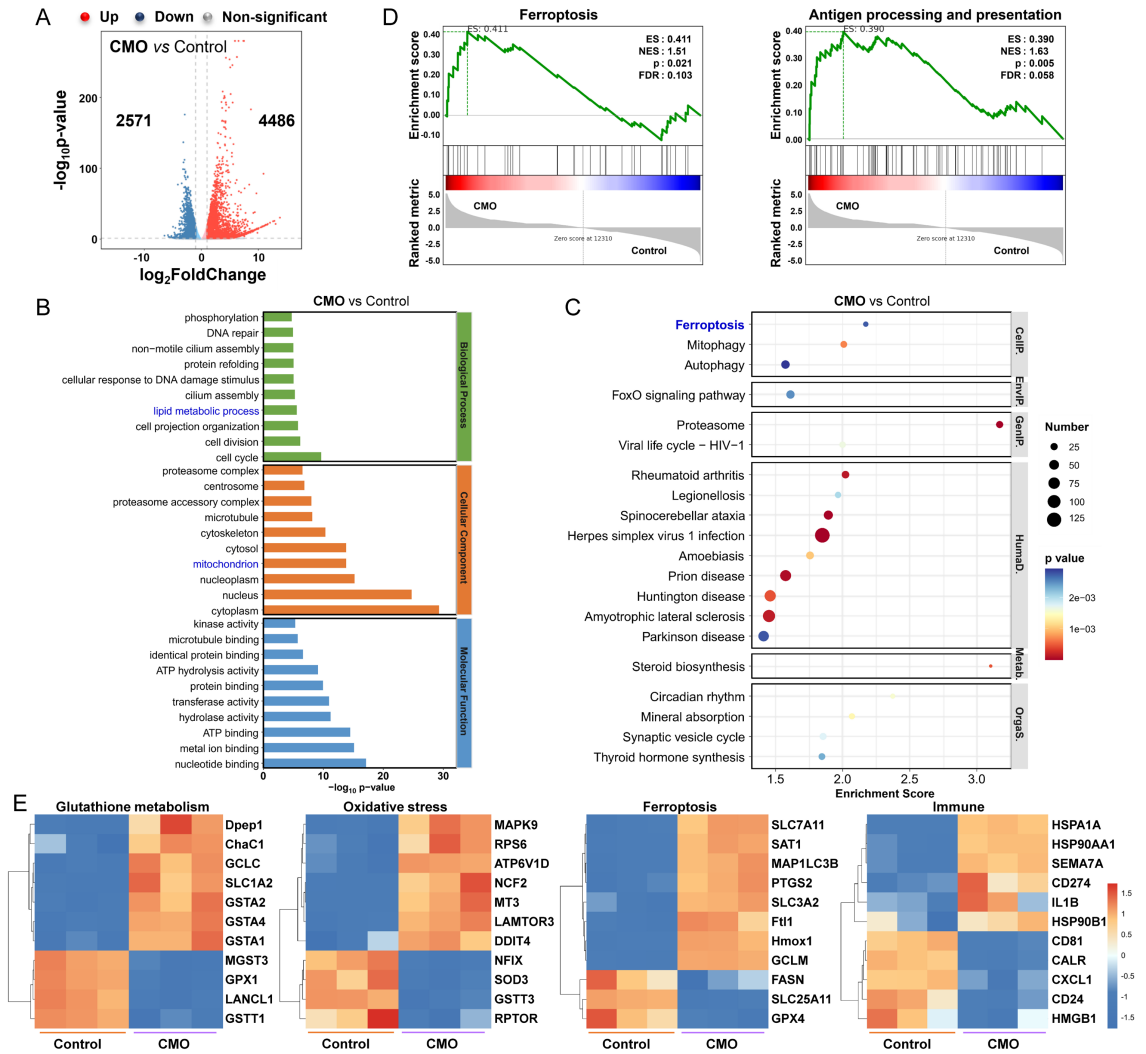
RNAseq analysis. (A) Volcano plot showing the DEGs in 4T1 cells treated with CMO (10 μg/mL) for 18 h. Standard: |log_2_ FC| >1; q value <0.05. (B) GO term analysis (top 10 in total of upregulated and downregulated from each classification) based on RNAseq after CMO treatment. (C) KEGG term analysis (top 20 upregulated) based on RNAseq after CMO treatment. (D) GSEA revealing negative and positive enrichment of CMO altered genes in various pathways. ES: enrichment score. NES: normalized enrichment score. (E) Heat map of DEGs associated with glutathione metabolism, oxidative stress, ferroptosis, and immune in 4T1 cells after CMO treatment.

To confirm the fundamental therapeutic mechanism of CMO, RNA-sequence (RNA-seq) on 4T1 cells treated with CMO (10 μg/mL) were performed. According to Figures 6A and S24, the heatmap and volcano plot revealed 7057 differentially expressed genes (DEGs) (|log2 FC| >1; q value <0.05) between the control and CMO-treated groups, with 4486 genes up-regulated and 2571 genes down-regulated. Gene ontology (GO) enrichment analysis showed that CMO mainly influences lipid metabolic process, mitochondrion, cell cycle, cell division, ATP hydrolysis activity, etc (Figure 6B, S25, S26). Kyoto Encyclopedia of Genes and Genomes (KEGG) enrichment analysis confirmed that CMO mainly influences ferroptosis pathway in 4T1 cells along with fatty acid biosynthesis/degradation and cysteine/methionine metabolism (Figure 6C, S27). Wiki Pathways annotations analysis further confirmed the substantial impact on genes related to the oxidative stress response and metabolic processes (Figure S28). Gene set enrichment analysis (GSEA) of differentially expressed genes (DEGs) was performed to elucidate the underlying biological mechanisms. The analysis identified that CMO treatment significantly altered genes associated with ferroptosis, as well as antigen processing and presentation. (Figure 6D).

To determine the specific mechanism of CMO, the related heat maps of DEGs were generated (Figure 6E). Due to the distinct GSH consumption of CMO, the intracellular GSH metabolism related genes showed evident variations in expression including GPX1, Gstt1 46, GSTA4 47, and ChaC1 48 CMO treated group exhibited various DEGs, including SOD3 49, NCF2 50, NFIX 51, MT3 52 and other oxidative stress-related genes, indicating oxidative stress resulted from multienzymatic reactions for inducing changes in related pathways. Oxidative stress in cancer cells, along with the activation of negative feedback loops, may lead to the upregulation of HMOX1 53, 54, SLC7A11 55 and SLC3A2 56 as well as downregulation of GPX4 and FASN 57. Those DEGs are highly associated with lipid and cysteine metabolism which suggested the emergence of ferroptosis. The upregulation of HSPA1A, HSP90AA1, HSP90B1, HMGB1, and CALR suggested the inducement of ICD 58. Upregulation of immuno-promoting CD81/CD55 and PD-L1 transcription promoter CD274 also stimulated immunity responses and enhanced the inhibitory effect on cancer cells 59. The results validated the CMO treatment could effectively reduce GSH levels, enhance oxidative stress of cancer cells, thereby inducing immunogenic ferroptosis.

### MR imaging guided therapeutic effects of CMO

**Figure 7:**
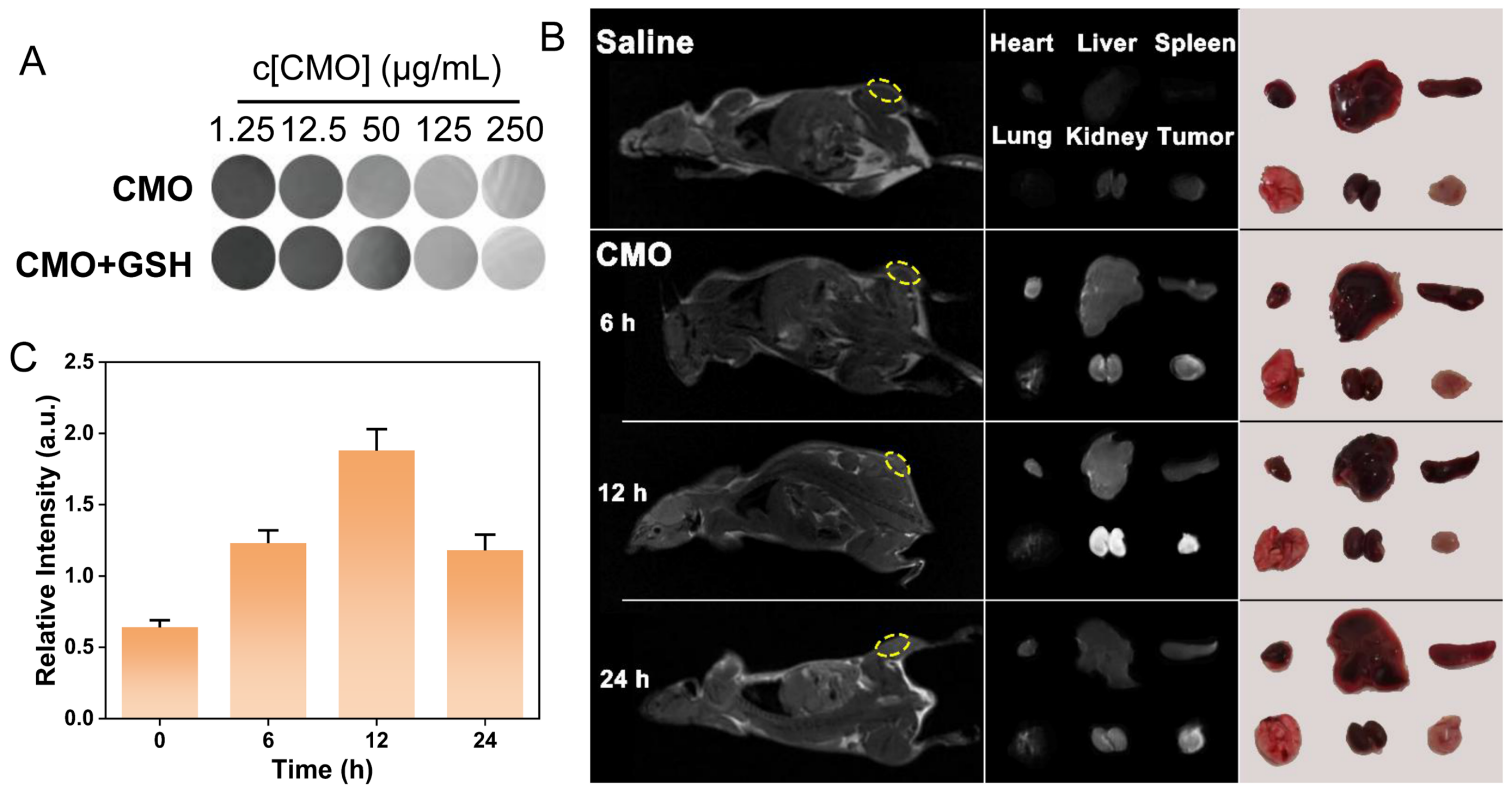
MR imaging capacity. (A) ^1^H *T*_1_-weighted MR images of various concentrations of CMO with or without GSH (8 mM). (B) *In vivo T*_1_-weighted MR images of tumor-bearing mice after i.v. injection of CMO. Individual *ex vivo* MR images and pictures of organs and tumors dissected from mice after different periods of i.v. injection. (C) The relative *T*_1_-weighted intensity of the selected tumor region in yellow circle in (B).

The 4T1 tumor-bearing mice models were established to validate the tumor inhibition efficiency of CMO *in vivo*. Considering paramagnetic Mn-based nanomaterials have been extensively used as *T*_1_-weighted MRI contrast agents in tumor diagnosing,^60^ the imaging capability of various concentrations of CMO was evaluated in the absence or presence of GSH (8 mM). The *T*_1_-weighted signal intensity of CMO was significantly enhanced with the increased concentrations of CMO (Figure 7A). The dose-dependent contrast of CMO maintain after incubation with GSH, suggesting the imaging capacity to assess *in vivo* accumulation and metabolism (Figure S29). 4T1-tumor-bearing BALB/c mice was injected with CMO *via* tail intravenous (i.v.) administration, followed by MR imaging at 6, 12, and 24 h. The *T*_1_-weighted signal intensity at the tumor sites (yellow circle) in mice was significantly enhanced at 6 h after injection, and reached maximum at 12 h (Figure 7B-C, S30). Then the *T*_1_ signal intensity was declined after 24 h of injection. To further investigate the time-dependent nanozyme distributions, the heart, liver, spleen, lung, kidney and tumor tissues were collected for MR imaging (Figure 7B). The distinct MR signals in kidney and tumor peaked 12 h after injection and then reduced after 24 h. A transient signal was also observed in the lung region at 12 h post-injection, which is consistent with the normal biodistribution pattern of *i.v.* administered nanoparticles via the mononuclear phagocyte system. This signal returned to baseline by 24 h, and no adverse pulmonary effects were noted throughout the study period. It indicated the rapid metabolism *via* kidney of CMO for enhancing its biosafety.

**Figure 8:**
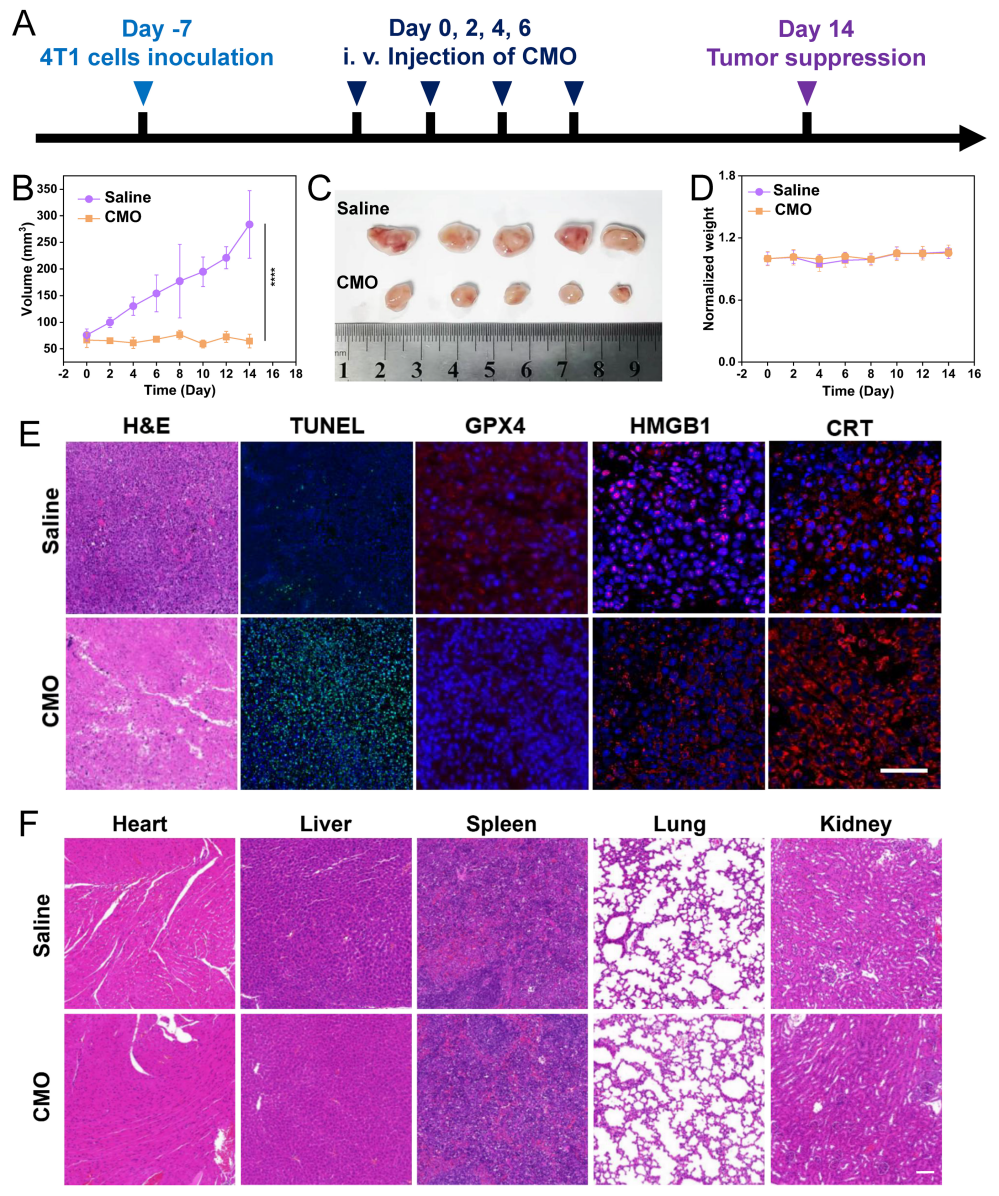
*In vivo* anticancer performances. (A) Schematic illustration of model establishment and treatment. (B-D) *In vivo* antitumor therapeutic effect: tumor volume recorded from Day 0 to Day 14 (B), photos of tumors dissected from mice after 14 days (C), and recorded body weight (D) of 4T1-tumor-bearing mice upon treatments of saline or CMO. All data are presented as means ± SD (n = 5 independent experiments for (B-D). Significance between two groups was assessed by Paired Sample t-test. (E) Tumor sections with H&E, TUNEL, GPX4, HMGB1, and CRT staining after saline or CMO treatment. Scale bar = 200 μm. (F) H&E staining images of the heart, liver, spleen, lung and kidney collected from different treatment groups. Scale bar = 200 μm.

According to the 24 h *in vivo* metabolism period, the i.v. administration interval on 4T1-tumor-bearing BALB/c mice was determined as 48 h in the tumor treatment (Figure 8A). After 14 days and four i.v. administrations in total, significant tumor suppression was observed in the CMO group compared to the saline group, with negligible weight variations (Figure 8B,D). The collected tumor tissues showed distinct size reduction in CMO-treated mice (Figure 8C). The mice were sacrificed after all treatments, the tumor suppressive effect of the CMO were additionally assessed through GPX4 and Ki67 antibody staining, terminal deoxynucleotidyl transferase-mediated deoxyuridine triphosphate nick-end labeling (TUNEL) staining and hematoxylin and eosin (H&E) staining (Figure 8E). In contrast to the saline group, the tumor sections from the CMO administration groups displayed increased cell necrosis as indicated by H&E and TUNEL staining, demonstrating more pronounced tumor tissue damage (Figure 8E). Notably, the widespread positive TUNEL fluorescent signals explicitly confirmed the occurrence of severe DNA fragmentation, a hallmark of apoptosis *in vivo*. This demonstrated that within the complex TME, the oxidative stress induced by ultrafast GSH depletion and ROS generation triggered a potent crosstalk between ferroptosis and apoptosis, synergistically amplifying the overall antitumor tissue damage. Ki67 staining revealed no significant cellular proliferation in the CMO administration group compared to the control group (Figure S31). The conspicuous downregulation of GPX4 was observed in tumor treated with CMO, verifying the ferroptosis *in vivo*.The induction of ICD by CMO treatment was further examined *ex vivo*, building on the *in vitro* findings of increased CRT expression and HMGB1 secretion. Tumor samples were subjected to immunofluorescence (IF) staining to assess CRT exposure and HMGB1 release. Consistent with the *in vitro* results, the CMO-treated group showed significantly higher levels of CRT surface presentation and HMGB1 extracellular release compared to the control group (Figure 8E). The main organs, such as heart, lungs, spleen, liver, and kidneys, were harvested for H&E staining which displayed no visible damage (Figure 8F). In addition, fresh blood was collected for routine blood after all treatments (Figure S32). Compared with the saline group, the hemolysis test showed that the majority of hematological index abnormalities were alleviated.

**Figure 9:**
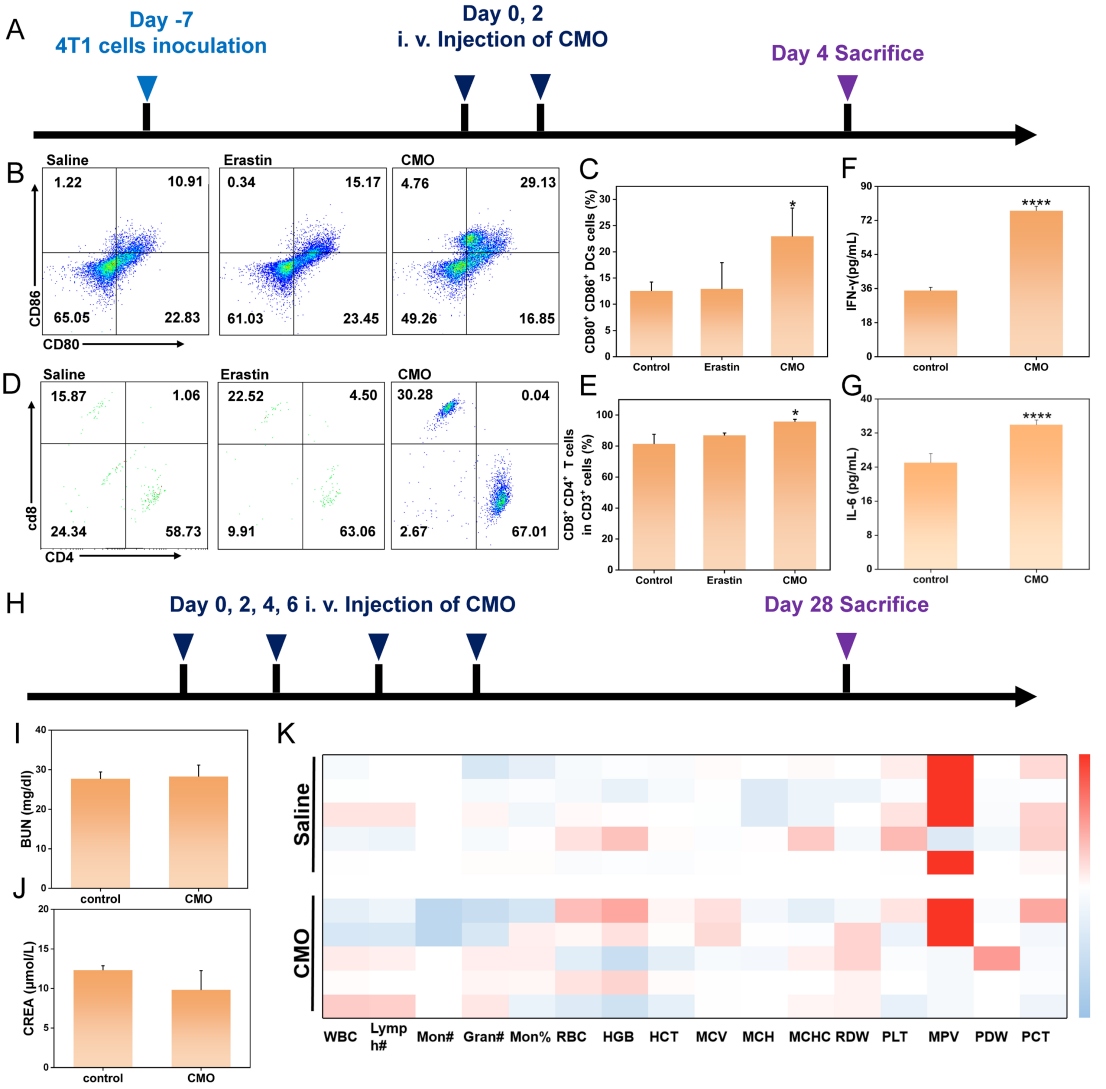
*In vivo* immunotherapeutic effects and the long-term safety. (A) Schematic illustration of experimental process of *in vivo* antitumor immunity. (B) Flow cytometry analysis of mature DCs of tumors after saline, Erastin and CMO treatment. (D) Flow cytometry analysis of CD4^+^ T cells and CD8^+^ T cells of tumors after saline, Erastin and CMO treatment (the CD4^+^ T cells and CD8^+^ T cells were gated on CD3^+^ T cells). (C, E) Quantitative analysis of the results in b,d. All data are presented as means ± SD (n = 3 independent experiments). Significance between two groups was assessed by Paired Sample t-test. (F, G) Quantity of inflammatory cytokines IL-6 and IFN-γ after different treatments. All data are presented as means±SD (n = 3 independent experiments). Significance between two groups was assessed by Paired Sample t-test. (H) Schematic illustration of experimental process of *in vivo* safety for 28 days. (I-J) Renal blood analysis in different groups of normal mice after 28 days of treatment. (mean ± SD, n = 5). (K) Hematological indexes of mice after i.v. injection with Saline and CMO for 28 days.

As antigen-presenting cells, dendritic cells (DCs) are essential for initiating and regulating both innate and adaptive immunity, thereby significantly promoting T cell proliferation. The induction of ferroptosis and immunogenic cell death (ICD) suggested their potential to trigger tumor-specific immune responses. The immune-activating capability of CMO was further confirmed by assessing DC maturation and T cell infiltration (Figure 9A). Following CMO treatment, the proportion of mature DCs within the tumor was approximately 2.67 times higher than that observed in the saline group (Figure 9B-C). This DC maturation in turn facilitated the infiltration of T cells into the tumor. Cytotoxic T lymphocytes (CTL, CD8^+^ T cells) along with helper T cells (CD4^+^ T cells) engage in vital functions in the direct combat toward cancer cells, which are essential for the regulation of adaptive immunities. CD4^+^ T cells and CD8^+^ T cells in CD3^+^ T cells displayed a distinct increase in the CMO groups from 81.39% to 95.76%, thereby revealing local immune response in the tumors (Figure 9D, 9E, S33-34). This profound local immune cell infiltration directly confirmed that the ferroptosis mode induced by CMO was highly immunogenic, activating a robust anti-tumor adaptive immune response. Notably, we also included Erastin as a positive control to benchmark the *in vivo* immune activation. The flow cytometry results demonstrated that CMO treatment induced a higher percentage of mature DCs (29.13%) and a more profound infiltration of CD8^+^ T cells (67.01%) compared to the classical ferroptosis inducer Erastin (15.17% and 63.06%, respectively) (Figure 9B-E). This comparison highlights the superior immunogenic efficacy of the CMO nanozyme in remodeling the immunosuppressive TME.

To further validate the systemic anti-tumor immune response triggered by ICD, we evaluated the levels of key inflammatory cytokines in the serum of the tumor-bearing mice. As shown in Figures 9F-G, the secretion of both IL-6 and IFN-γ in the CMO treatment group was significantly elevated compared to the control group. The robust systemic cytokine release perfectly corroborates our findings of enhanced T-cell infiltration. Together with the release of HMGB1 and CRT exposure, this completes a rigorous evidence chain, confirming that the CMO nanozyme successfully induces immunogenic cell death and translates it into a potent anti-tumor immunity.

To evaluate the long-term biosafety of CMO, healthy mice were intravenously administered CMO via the tail vein every two days for a total of four doses (on days 0, 2, 4, and 6), and were subsequently monitored until day 28 post-treatment (Figure 9H). At the 28-day endpoint, fresh blood samples were collected for complete blood count (CBC) and systemic serum biochemistry analyses (Figure 9K). Liver function was thoroughly assessed by measuring alanine aminotransferase (ALT), aspartate aminotransferase (AST), total bilirubin (TBIL), and total protein (TP) (Figure S35). Concurrently, kidney function was evaluated via blood urea nitrogen (BUN), uric acid (UA), and creatinine (CREA) levels (Figure 9I-J, S35). Notably, all evaluated parameters in the CMO-treated group remained strictly within normal physiological ranges and exhibited no statistically significant differences compared to the control group. These results indicate that the CMO nanozyme possesses favorable *in vivo* biocompatibility with no detectable long-term systemic toxicity.

## Conclusions

In summary, a Bir-type manganese oxide nanozyme CMO with ultrathin 2D morphology has been prepared through a facile one-pot hydrothermal method using non-precious metal elements Mn and Cu. CMO exhibited an ultrafast GSH depletion capacity within 1 min with its concentration of 20 μg/mL. CMO could also oxidize GSH with its own lattice oxygen in an oxygen-free path according to theoretical investigation, which provide GSH consumption capacity under hypoxia. CMO has been further confirmed with POD/CAT mimicking activities. CMO showed enhanced *in vitro* antitumor activity under hypoxia (IC_50_ 2.01 μg/mL) compared to that under normoxia (IC_50_ 47.77 μg/mL). The *in vitro* study also confirmed the activation of ferroptosis and induction of ICD which were caused by the accumulation of LPO through ROS boost, GSH oxidation and GPX4 downregulation. Moreover, CMO could be utilized as an MR imaging contrast agent for tumor diagnosis and treatment monitoring. *In vivo* anticancer activity and biosafety have been confirmed on a 4T1 tumor-bearing mouse model. Crucially, *in vivo* immunological evaluations confirmed that this aggressive ferroptotic pathway effectively triggers ICD. This process significantly promotes DC maturation and robust T-lymphocyte infiltration, successfully remodeling the immunosuppressive TME to execute potent systemic antitumor immunity. The study provides a non-precious metal-based Bir-type nanozyme platform to deliver ultrafast GSH depletion and immunogenic ferroptosis under hypoxia which is potent for imaging-guided tumor immunotherapy.

## Supplementary Material

Supplementary materials and methods, figures and table.
