# Supplementary material for: Glutathione depletion under hypoxia via a birnessite-type manganese oxide nanozyme inducing immunogenic ferroptosis for magnetic resonance imaging guided cancer therapy
Source: Theranostics. 2026 May 11;16(12):6783–802. doi: 10.7150/thno.129808 (PMC13232481; doi:10.7150/thno.129808)
Supplement: Supplementary file 1 — Supplementary materials and methods, figures and table. [file thnov16p6783s1.pdf]

Supporting Information

for

Glutathione depletion under hypoxia via a birnessite-type manganese oxide nanozyme inducing immunogenic ferroptosis for magnetic resonance imaging guided cancer therapy

Huilin Sun[#,a], Bo Li[#,a], Lin Qiu[#,a], Yufeng Zhao[a], Xin Wang[c], Chenxian Zhu[d], Haonan Zhang[b], Xiang Wang*[,b], Yang Bai*[,a], Jianhao Wang*[,a,c]

[a] School of Pharmacy & Affiliated Hospital of Changzhou University (Changzhou West Taihu Hospital), Changzhou University, Changzhou 213164, P. R. China.

[b] Department of Radiology, The Third Affiliated Hospital of Nanjing Medical University, Changzhou 213164, P. R. China.

[c] School of Medical and Health Engineering, Changzhou University, Changzhou 213164, P. R. China.

[d] Department of Gynecology, Changzhou Maternal and Child Health Care Hospital, Changzhou, 213000, P. R. China

[#] These authors contribute equally to this work.

E-mail: wx402@sohu.com (X. Wang), baiy@cczu.edu.cn (Y. Bai), minuswan@cczu.edu.cn (J. Wang)

1. Materials and instruments

The following reagents and instruments were obtained from commercial sources: Copper(II) nitrate trihydrate ($Cu(NO_3)_2 \cdot 3H_2O$), manganese(II) chloride tetrahydrate ($MnCl_2 \cdot 4H_2O$), and 1,2-distearoyl-sn-glycero-3-phosphoethanolamine-N-[methoxy(polyethylene glycol)2000] (DSPE-PEG2000) were supplied by Shanghai Ponsure Biotechnology Co., Ltd. Sodium hydroxide (NaOH), glutathione (GSH), 3,3',5,5'-tetramethylbenzidine (TMB), and hydrogen peroxide (30% H_2O_2) were procured from local vendors. Calcein-AM (acetoxymethyl ester of calcein), propidium iodide (PI), and 2',7'-dichlorodihydrofluorescein diacetate (DCFH-DA) were obtained from Beijing Solarbio Science & Technology Co., Ltd. The GSH and GSSG Assay Kit, as well as the Cellular Glutathione Peroxidase Assay Kit with NADPH, were purchased from Beyotime. The JC-1 Mitochondrial Membrane Potential Detection Kit was acquired from Servicebio. The following antibodies were obtained from the indicated commercial sources and used at the specified dilutions: β-Actin Rabbit Monoclonal Antibody (Beyotime, 1:1000), Recombinant Anti-Glutathione Peroxidase 4 antibody (Rabbit mAb) (Servicebio, 1:1000), HRP-labeled Goat Anti-Rabbit IgG(H+L) (Beyotime, 1:1000), Calreticulin Rabbit Monoclonal Antibody (Beyotime, 1:200), IgG (H+L), Cy3 conjugated Donkey Anti-Goat IgG (H+L) (Beyotime, 1:500), HMGB1 Rabbit Polyclonal Antibody (Beyotime, 1:500), and IgG (H+L), BeyoAb™ AF488-labeled Donkey Anti-Rabbit IgG(H+L) (Beyotime, 1:500).

X-ray diffraction (XRD) patterns were collected on a D8 Advance diffractometer from Rigaku Corporation (Japan). Scanning electron microscopy (SEM) images were captured using a Zeiss-Supra55 instrument, while transmission electron microscopy (TEM) images were acquired on a Philips Tecnai12 (Netherlands). Dynamic light scattering (DLS) and Zeta potential measurements were performed with a Malvern laser particle size analyzer (Malvern, UK). X-ray photoelectron spectroscopy (XPS) was conducted on a apparatus (Thermo Scientific ESCALAB 250). High-resolution mass spectrometry (HRMS) analysis was carried out using a Bruker mass spectrometer (models: Thermo Scientific Q Exactive and Agilent 7250). UV-vis absorption spectra were recorded on a spectrophotometer (Shimadzu UV-3600). Reactive oxygen species (ROS) levels were examined under a confocal laser scanning

microscope (CLSM, Ti2, Nikon Eclipse Co., Ltd., Japan). Flow cytometry data were obtained using a BD Accuri C6 Plus flow cytometer (Shanghai, China). Finally, magnetic resonance (MR) imaging was performed on a GE Discovery MR750 3.0T system.

2. Experimental section

2.1 Oxygen generation of CMO

A solution containing H_2O_2 (100 mM) and CMO (20 μg/mL) in PBS was prepared and stirred vigorously. The dissolved oxygen concentration in the mixture was recorded every two minutes using a portable dissolved oxygen meter. Using a similar approach, the effect of pH (5.5 and 7.4) on the oxygen production capability of CMO was also assessed.

2.2 In vitro assessment of $^1O_2$ generation

Using 1,3-diphenylisobenzofuran (DPBF) as a probe, the formation of $^1O_2$ was monitored by UV-Vis spectrometry. DPBF (0.1 mM) was mixed with CMO (20 μg/mL) in the presence or absence of H_2O_2 (20 mM). Then the absorbance at 405 nm at different reaction time points was determined by UV-Vis spectrometer. The control experiment was performed without the addition of CMO keeping the DPBF concentration consistent with the above.

2.3 Electron Spin Resonance (ESR) Measurements

To measure •OH generation *via* ESR, CMO (100 μg/mL) was incubated with H_2O_2 (100 mM) for 5 min in PBS solution (pH = 4). Then 5,5-dimethyl-1-pyrroline-N-oxide (DMPO) (100 mM) was added to the above solution, and the ESR signal was recorded by ESR spectrometer.

To measure $^1O_2$ generation *via* ESR, CMO (250 μg/mL) incubated with 2,2,6, 6-tetramethyl-4-piperidone hydrochloride (TEMP) (100 mM) for 10 min in H_2O, and the ESR signal was recorded by ESR spectrometer.

2.4 In vitro fluorescence detection of ROS

A mixture consisting of 500 μL of DCFH-DA ethanol solution (1.0 mM) and 2 mL of aqueous NaOH (10 mM) was stirred vigorously for 30 minutes. The hydrolysis was then terminated by adding 10 mL of

PBS at pH 4, yielding a DCFH solution. Subsequently, 10 μL of DCFH (25 μM) and 40 μL of CMO (1.0 mg/mL) were introduced into 200 μL of PBS (pH 4) either in the presence or absence of 100 μM H_2O_2. Meanwhile, the influence of GSH on ROS generation was examined using the same procedure, with the addition of 20 mM GSH. The fluorescence emission of each sample was measured at 522 nm upon excitation at 495 nm. Solutions containing only H_2O_2 or only CMO served as controls.

2.5 Quantification of the Internalization Efficiency of CMO into 4T1 Cells

The internalization efficiency of CMO in 4T1 cells was determined by ICP-MS. 4T1 cells were seeded in a six-well plate (3 × 10^5 cells/well), and incubated overnight to allow for adhesion. Then different concentrations of CMO (0, 1 and 10 μg/mL) were added into the plate for incubation for 6 and 24 h respectively. The culture medium was removed and the cells were washed with PBS solution for three times. Subsequently, the cells were separated and washed three times with PBS solution. The obtained cells were lysed with cell lysate, and the supernatant was collected by centrifugation after full lysis, and the contents of Cu and Mn in the samples were analyzed by ICP-MS.

2.6 Annexin V-FITC/PI Apoptosis Assay

The Annexin V-FITC/PI apoptosis assay was carried out by flow cytometry using a commercial kit. Briefly, 4T1 cells were added to a six-well plate at a density of 1.5 × 10^5 cells/well, and left standing overnight for adhesion. Then, different concentrations of CMO (0, 1, 10 μg/mL) were added for an incubation of 6 h. According to the instructions in the kit, apoptosis of the cells were analyzed.

2.7 Cell Cytotoxicity

The cytotoxicity of CMO was evaluated using the standard MTT assay. 4T1, MCF-7, and 3T3 cells were exposed to various concentrations of CMO for 24 hours. After adding MTT solution to each well and incubating for another 4 hours, the resulting blue formazan crystals were dissolved in 100 μL of DMSO. Cell viability was then determined by measuring the absorbance at 490 nm. Cytotoxicity under hypoxic conditions was also confirmed.

4T1 cells were added to a 96-well plate at a density of 1 × 10^4 cells/well, and left standing overnight for adhesion. The supernatant was discarded, and the cells were pre-treated with rescue agents

(GSSG/NAC/AA) for 2 hours, followed by the addition of CMO (10 μg/mL), CMO + GSSG, CMO + NAC, CMO + AA, GSSG (0.5 mM), NAC (2 mM), and AA (100 μM) respectively. 4T1 cells were incubated with various treatments for 24 h under normoxia and hypoxia condition. Following the addition of MTT solution to each well and a 4-hour incubation period, the resulting blue formazan product was solubilized in 100 μL of DMSO. Cell viability was then assessed by measuring the absorbance at 490 nm..

4T1 cells/MCF-7 cells were seeded in 96-well plates at a density of 1×10^4 cells per well and incubated overnight. Cells were pre-incubated with Fer-1 (1 μM) for 2 h, followed by treatment with CMO (10 μg/mL) or Erastin (20 μM) alone or in combination with Fer-1 for 24 h. The experimental groups included control, Fer-1 alone, CMO alone, Fer-1+CMO, Erastin alone, and Fer-1+Erastin. After treatment, 10 μL of MTT solution (5 mg/mL) was added to each well and incubated for 4 h. The supernatant was then removed, and the formazan crystals were dissolved in 100 μL DMSO. The viability of cells was evaluated by determining the absorbance at 490 nm.

A live/dead cell double staining kit was used to distinguish the live and dead cells after different treatments. Firstly, 4T1 cells (1.5×10^5 cells per well) were cultivated in 24-well plates overnight. Afterwards, the cells were treated with different concentrations of CMO (0, 5, 20 μg/mL). After incubation for next 24 h, the cells were incubated with 250 μL of the mixture of diluted Calcein-AM solution (1:1000) and diluted propidium iodide (PI) solution (1:1000) for 60 min and imaged by the fluorescence microscope.

2.8 Cellular ROS Assay

The level of intracellular ROS generated by CMO was evaluated using flow cytometry with the DCFH-DA probe. 4T1 cells were seeded into six-well plates at a density of 1.5×10^5 cells per well and allowed to settle overnight. Afterward, varying concentrations of CMO (0, 1, and 10 μg/mL) were added, and the cells were incubated for 24 hours. The culture medium was then removed, and the cells were detached and washed three times with PBS solution. The fluorescent probe DCFH-DA (10 μM) was added followed by incubation in the dark at 37 ºC for 30 min. Flow cytometry was used to detect the fluorescence intensity of the treated cells under the excitation of 488 nm and the emission of 530 nm.

4T1 cells (3.0×10^5) were inoculated into a confocal dish. 4T1 cells were incubated with different concentrations (0, 5, 20 μg/mL) of CMO for 4 h, and washed with PBS solution. The fluorescent probe DCFH-DA (10 μM) was added to the cells and incubated in the dark at 37 ºC for 30 min. After PBS washing, the fluorescence of the sample was determined by CLSM (excitation: 488 nm).

2.9 Western Blot

The protein level of glutathione peroxidase 4 (GPX4) was determined using Western blot analysis. In brief, 4T1 cells were seeded at a density of 2×10^6 cells per 100 mm culture dish and left to attach overnight. The cells were then treated with varying concentrations of CMO (0, 5, 10, and 20 μg/mL) for 24 hours. After incubation, the harvested cell lysates were resolved on 15% SDS-polyacrylamide gels and then electrotransferred onto polyvinylidene fluoride membranes. The protein of interest was immunodetected using specific primary antibodies, followed by incubation with HRP-conjugated anti-rabbit secondary antibodies. Chemiluminescent signals were visualized using a luminol-based enhanced chemiluminescence HRP substrate.

2.10 Mitochondrial Membrane Potential Assay

The assessment of mitochondrial membrane potential loss was performed via flow cytometry using a commercially available kit (Servicebio, Wuhan, China). In brief, 4T1 cells were seeded at a density of 3.0×10^5 cells per well in six-well plates and allowed to attach overnight. Subsequently, the cells were exposed to different concentrations of CMO (0, 5, and 20 μg/mL) for 24 hours. All subsequent operations were carried out according to the kit manufacturer’s protocol. The resulting fluorescence signals were measured by flow cytometry and the data were analyzed accordingly.

2.11 Cellular Lipid Hydroperoxide Assay

The level of cellular lipid hydroperoxides was evaluated using flow cytometry with the BODIPY581/591-C11 probe. In brief, 4T1 cells were seeded at 3.0×10^5 cells per well in six-well plates and left to adhere overnight. The cells were then treated with various concentrations of CMO (0, 5, and 20 μg/mL) for 12 hours. All subsequent steps were carried out according to the probe manufacturer’s protocol. Fluorescence intensities were measured by flow cytometry and the resulting data were analyzed.

2.12 Cellular malondialdehyde (MDA) assay.

4T1 cells were placed into six-well plates at a density of 3×10^5 cells per well and cultured for 24 hours. Following the specified treatments, the cells were collected and rinsed with PBS. Subsequently, the malondialdehyde (MDA) levels in the treated cells were determined by using a Lipid Peroxidation MDA Assay Kit (Beijing Solarbio Science & Technology Co., Ltd., China) according to the standard procedures.

2.13 Cellular immunofluorescence

4T1 cells (5×10^5) were placed into a confocal dish overnight and then treated with either PBS solution or 10 μg/mL CMO for 12 hours. After treatment, the cells were rinsed three times with PBS and fixed with 4% paraformaldehyde for 15 minutes. Following fixation, the cells were washed three more times with PBS and permeabilized with 0.1% Triton X-100 for 15 minutes at room temperature. The cells were blocked with BSA for 30 minutes at room temperature after carefully removing the permeabilization solution. And then the cells were treated overnight at 4 °C with primary antibodies targeting HIF-1α, CRT, or HMGB1 following three PBS washes, respectively. The next day, after three additional PBS rinses, the cells were incubated for 30 minutes at room temperature with either Cy3- or FITC-conjugated goat anti-rabbit secondary antibodies. Finally, the nuclei were counterstained with DAPI-containing mounting medium.

2.14 Intracellular ATP level

4T1 cells were treated with various concentrations of CMO (0, 5, 10, and 20 μg/mL) for 24 hours. Afterward, the cells were rinsed, dissociated, and counted using a cell counter. For each concentration group, an equal number of cells were combined with 300 μL of lysis solution and homogenized for 5 minutes. Then supernatants were collected by centrifugation (12000 rpm, 10 min) for further processing following the manufacturer's instructions. ATP contents of above supernatants were detected on a microplate reader by measuring the luminescence.

2.15 RNA-seq analysis

4T1 cells were seeded in 10 cm culture dishes for 18 h and treated with CMO (10 μg/mL). After treatment, the cells were harvested and total RNA was extracted by TRIzol Reagent (Beyotime). The samples were then sent to OE Biotech Co., Ltd. (Shanghai, China) for RNA sequencing (RNA-seq).

2.16 quantitative reverse transcription Polymerase Chain Reaction

4T1 cells were grown overnight in 6 cm dishes at a density of 1×10^6 cells per dish. They were then incubated for 18 hours either with or without 10 μg/mL CMO. After these treatments, total RNA was isolated using TRIzol reagent. RNA concentrations were determined, and quantitative PCR (qPCR) was carried out by Wuhan Servicebio Technology Co., Ltd., following standard protocols.

2.17 Antitumor immunity in vivo

For the establishment of a subcutaneous tumor model, 4T1 cells (1.5×10^7) were randomly divided, resuspended in PBS, and subsequently injected into the right dorsal flank area of the mice. Once the tumor volume reached approximately 100 mm^3, the tumor-bearing mice were randomly divided into three treatment groups (n = 3 mice per group). The mice received intravenous injections as follows: saline (0.9% NaCl) for group 1; Erastin (1.5 mg/kg, prepared from a 10 mM stock solution in DMSO and diluted with a vehicle consisting of 40% PEG400, 5% Tween-80, and 45% saline) for group 2; and CMO (4 mg/kg) for group 3. After two rounds of treatment, the mice were sacrificed, and the harvested tumor tissues were sent to Wuhan Servicebio Technology Co., Ltd.

Additionally, the six mice that were implanted with tumors by the 4T1 cells were also blood sampled after four administrations of CMO for the determination of cytokines (IL-6 and IFN-γ).

2.18 In vivo biological safety

Female BALB/c mice (6-8 weeks old, ~20 g) were purchased from the Changzhou Cavens Biological Technology Co., Ltd. (Changzhou, Jiangsu, China). Mice were maintained with SPF food and water for 2 week. The mice were randomly divided into two groups as follows: 1) Saline; 2) CMO, and used for biosafety assessment. For the intravenous injection studies, mice received intravenous injections of either CMO (4 mg/kg) or saline every two days, for a total of four administrations. On the 28-th day, the mice were sacrificed. Fresh blood from BALB/c mice treated with saline and CMO (4 mg/kg) was collected

for serum biochemistry assay and complete blood panel analysis, which were conducted in Wuhan servicebio technology CO.,LTD.

2.19 Blood Analysis

Fresh blood from BALB/c mice treated with saline and CMO (4 mg/kg) was collected for serum biochemistry assay and complete blood panel analysis, which were conducted in NanJing YouMeng Biology Science and Technology Co., Ltd.

3. Computational details

All density functional theory (DFT) calculations were performed using the CP2K software package.[S1] A mixed basis set of Gaussian and plane waves was employed throughout. Core electrons were described by norm-conserving Goedecker-Teter-Hutter pseudopotentials,[S2] while valence electron wavefunctions were expanded using a double-zeta basis set augmented with polarization functions[S3], together with an auxiliary plane-wave basis set at a cutoff energy of 360 eV. The Perdew-Burke-Ernzerhof (PBE)[S4] generalized gradient approximation was adopted for the exchange-correlation functional. Geometry optimizations were carried out using the Broyden-Fletcher-Goldfarb-Shanno (BFGS) algorithm, with a self-consistent field (SCF) convergence criterion of 1.0×10^{-6} a.u. To account for long-range van der Waals dispersion interactions between the adsorbate and the framework, the DFT-D3[S5] correction including an empirical damped potential term was added to the energies obtained from the exchange-correlation functional in all calculations.

The Gibbs free energy change for each elementary step was computed at 298.15 K and defined according to standard formulas, as follows:

$$\Delta G = \Delta E_{DFT} + \Delta E_{ZPE} - T\Delta S$$

where ΔE represents the electronic energy difference obtained from CP2K calculations, ΔE_{ZPE} denotes the zero-point energy difference, and $T\Delta S$ corresponds to the entropy change term. For each reaction intermediate, the values of ΔE_{ZPE} and $T\Delta S$ were determined using the following equations,

$$E_{ZPE} = \frac{1}{2} \sum_i h v_i$$

$$TS = \sum_i h v_i \left(\frac{1}{e^{h v_i / k_B T}} \right) - k_B T \sum_i \ln \left(1 - e^{-h v_i / k_B T} \right)$$

where h, v_i, and k_B are Planck's constant, vibrational frequencies, and Boltzmann constant, respectively. For the vibrational frequency calculations, only the adsorbates were considered, assuming negligible contributions from the catalyst framework.

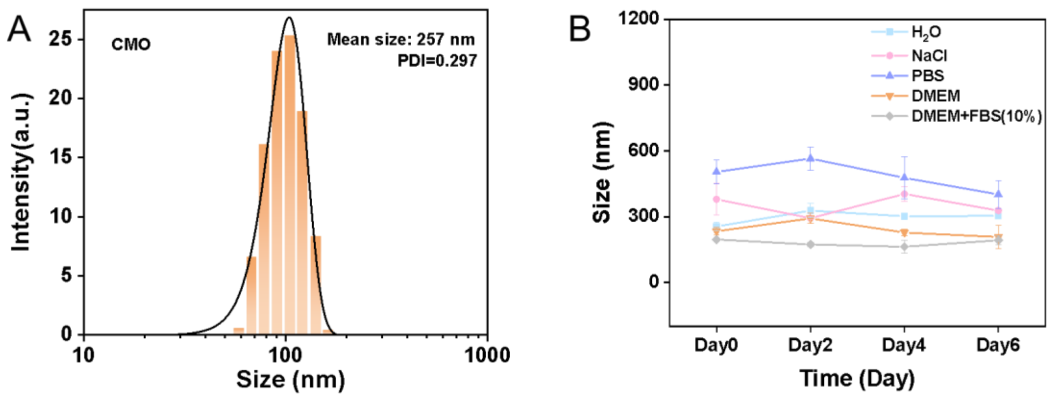

Figure S1. (A) The hydrodynamic size distribution and polydispersity index (PDI) of CMO. (B) The hydrodynamic size of CMO in Water, NaCl, PBS, DMEM, DMEM+10%FBS for different days, n=3.

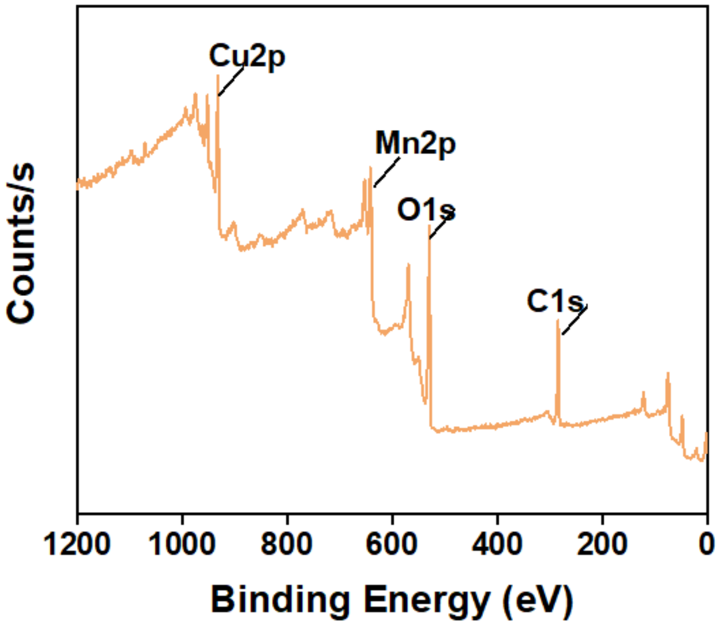

Figure S2. XPS survey spectra of CMO.

Table S1 The GSH depletion performances of CMO and recently reported nanozymes or nanomaterials.

Material	Metals	Material concentration	GSH concentration (mM)	Time upon fully depletion	References
**$CuMnO_2$**	**Cu, Mn**	**20 μg/mL**	**0.2**	**1 min**	**This work**
FNS	Fe	20 μg/mL	0.5	30 min	*Chin. Chem. Lett.* **2026***, 37,* 111693
CuP	Cu	50 μg/mL	0.5	3 h	*Bioact. Mater.* **2026,** *62,* 64–80
h^3-FNCs	Fe	10 μg/mL	0.3	30 min	*Nano-Micro Lett.* **2025**, *17*, 32.
CCFOB	Fe, Cu	166.7 ppm	0.133	40 min	*Chem. Eng. J.* **2025**, *507,* 160645.
DWN	W, Fe	200 μg/mL	1	240 min	*Chem. Eng. J.* **2025**, *509, 161353*
MMP	Mo, Mn	100 μM	1	54 h	*Bioact. Mater.* **2024**, *31*, 53–62
MLSLF	Fe	100 μg/mL	10	>24 h	*Chem. Eng. J.* **2024**, *479*, 147464
FeNv/CN	Fe	400 μg/mL	0.15	12 h	*Biomaterials* **2024**, *305*, 122446
VS_2-PEG	V	100 μM	1	36 h	*ACS Nano* **2023**, *17*, 17105−17121
$Cu_{2-x}Se$ nanozymes	Cu, Se	100 μg/mL	2	200 min	*Nano Today* **2024**, *54,* 102113
FNP MPNs	Fe	100 μg/mL	10	40 min	*Small* **2023**, *19*, 2207825
MoO_X-PEG NPs	Mo	25 μg/mL	1	48 h	*Angew. Chem. Int. Ed.* **2023**, *62*, e202215467
UiO@Mn_3O_4	Mn	150 μg/mL	1	12 h	*Theranostics* **2023**, *13*, 4121-4137
CuP_3@PPDG	Cu	200 μg/mL	1	48 h	*ACS Nano* **2023**, *17*, 11492−11506
MnOOH	Mn	200 μg/mL	10	>200 min	*J. Am. Chem. Soc.* **2023**, *145*, 5803−5815

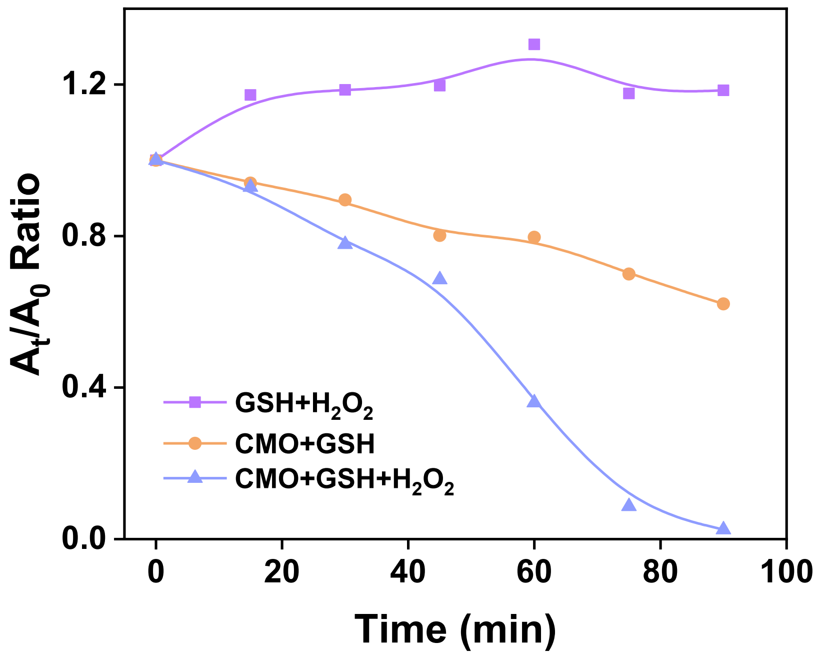

Figure S3. The ratio (A_t/A_0) of the absorbance at 412 nm of 0.1 mM DTNB reacted with CMO /GSH/H_2O_2 under different conditions. 5,5'-dithiobis(2-nitrobenzoic acid) (DTNB) was used for exploring the GSH depletion capacity of CMO. DTNB could be reduced by GSH to produce 5-thio-2nitrobenzoic acid (TNB, yellow product), which could be determined by absorbance at 412 nm. (purple: GSH+H_2O_2; orange: CMO+GSH; blue: CMO+GSH +H_2O_2)

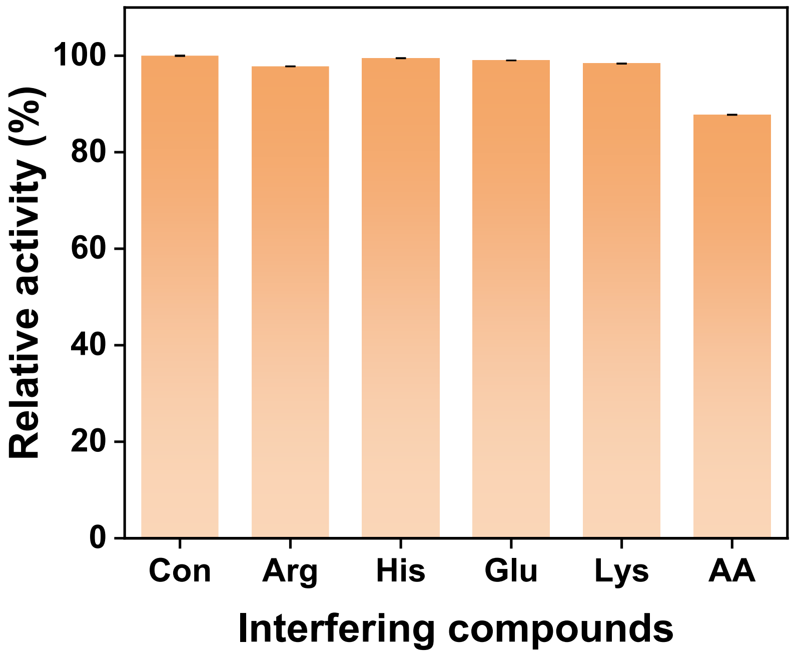

Figure S4. Selective oxidation of GSH by CMO versus other interfering compounds: none (Con), arginine(Arg), histidine (His), glucose (Glu), lysine (Lys), and ascorbic acid (AA) under a physiological environment. The ratio $[(A_0\text{-}A_t)/A_0]$ of the absorbance at 412 nm of 0.1 mM DTNB with the mixture of 0.2 mM GSH and 20 μg/mL CMO upon addition of various reagents.

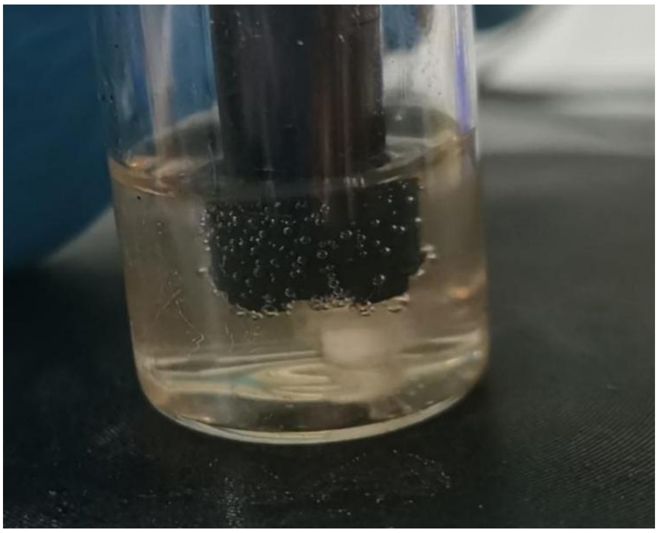

Figure S5. The optical picture of O_2 generation process.

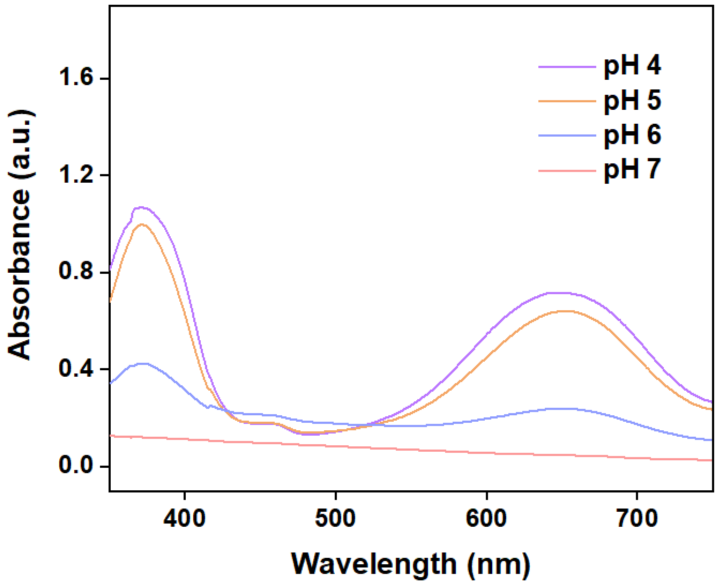

Figure S6. UV absorption of 0.6 mM TMB reacted with 10 μg/mL CMO and 0.66 mM H_2O_2 in different pH.

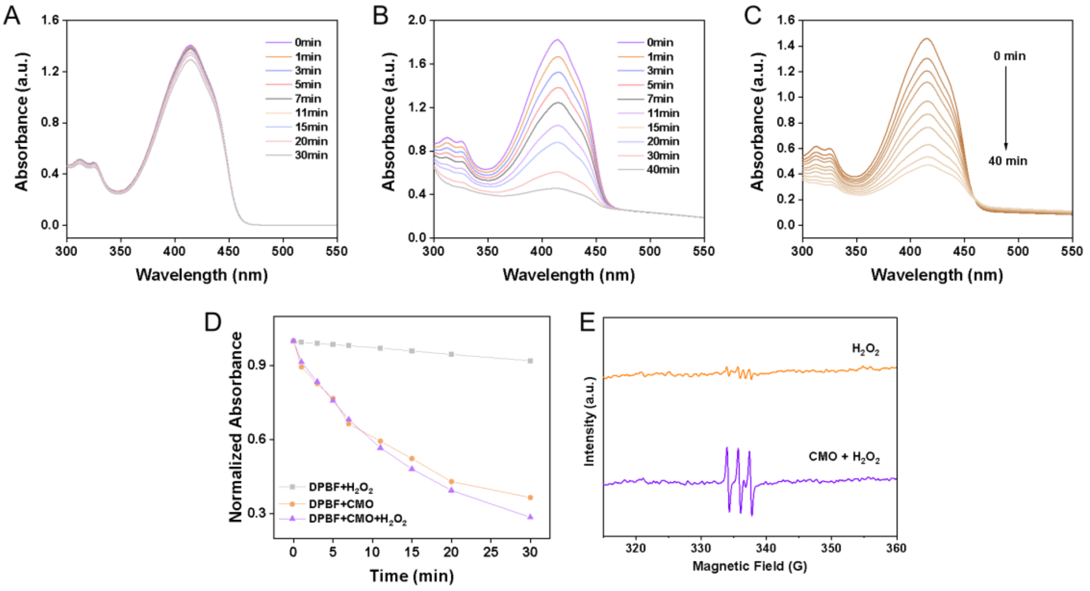

Figure S7. (A-C) Time-lapse UV-vis absorption of 0.1 mM DPBF (A), 0.1 mM DPBF mixed with 20 mM H_2O_2 and 20 μg/mL CMO (B) and 0.1 mM DPBF reacted with 20 μg/mL CMO (C). (D) The ratio of absorbance (A_t/A_0) recorded at 412 nm of 0.1 mM DPBF or after reaction with CMO or CMO and H_2O_2 in (A-C). (E) ESR signals of H_2O_2 in the absence (orange) and presence of 20 μg/mL CMO (purple) using TEMPO as the trapping agent.

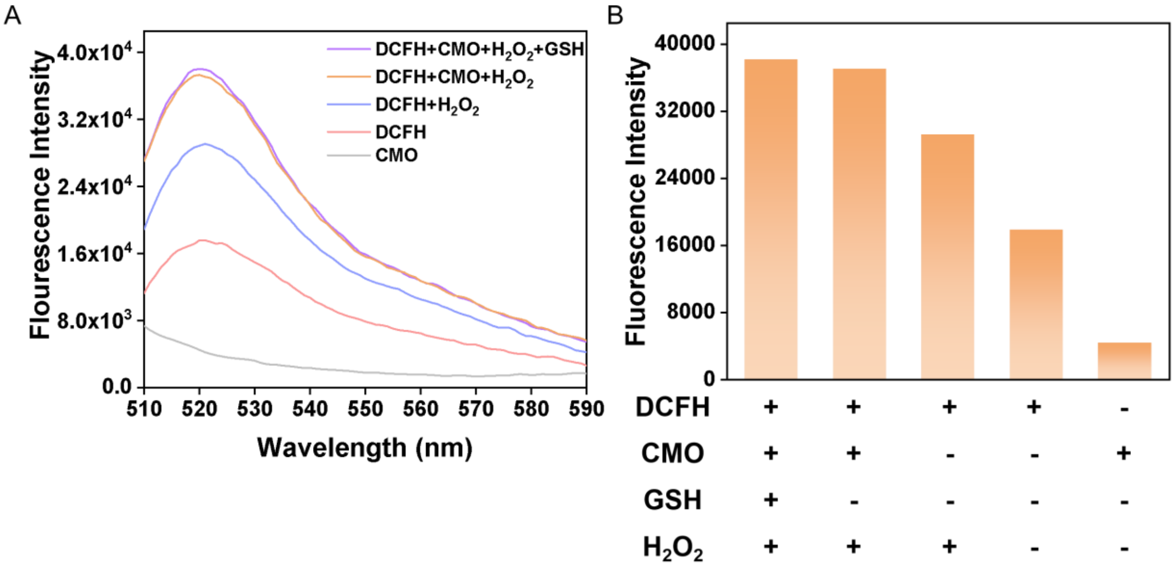

Figure S8. (A) Fluorescence spectra and (B) corresponding intensity at 520 nm of DCFH under different circumstances.

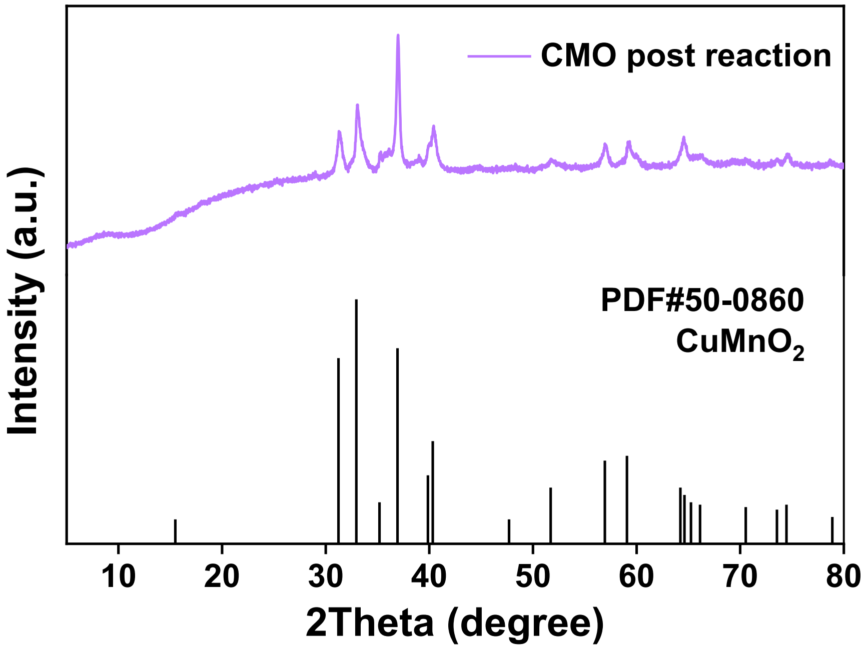

Figure S9. XRD pattern of CMO after reaction with GSH.

Table S2. The diffraction peak shift of CMO after reaction with GSH.

CMO/facets	Pristine/^{o}	Post-reaction/^{o}
002	31.42	31.28
200	33.20	33.04
$11\bar{1}$	37.04	36.98
111	40.62	40.42

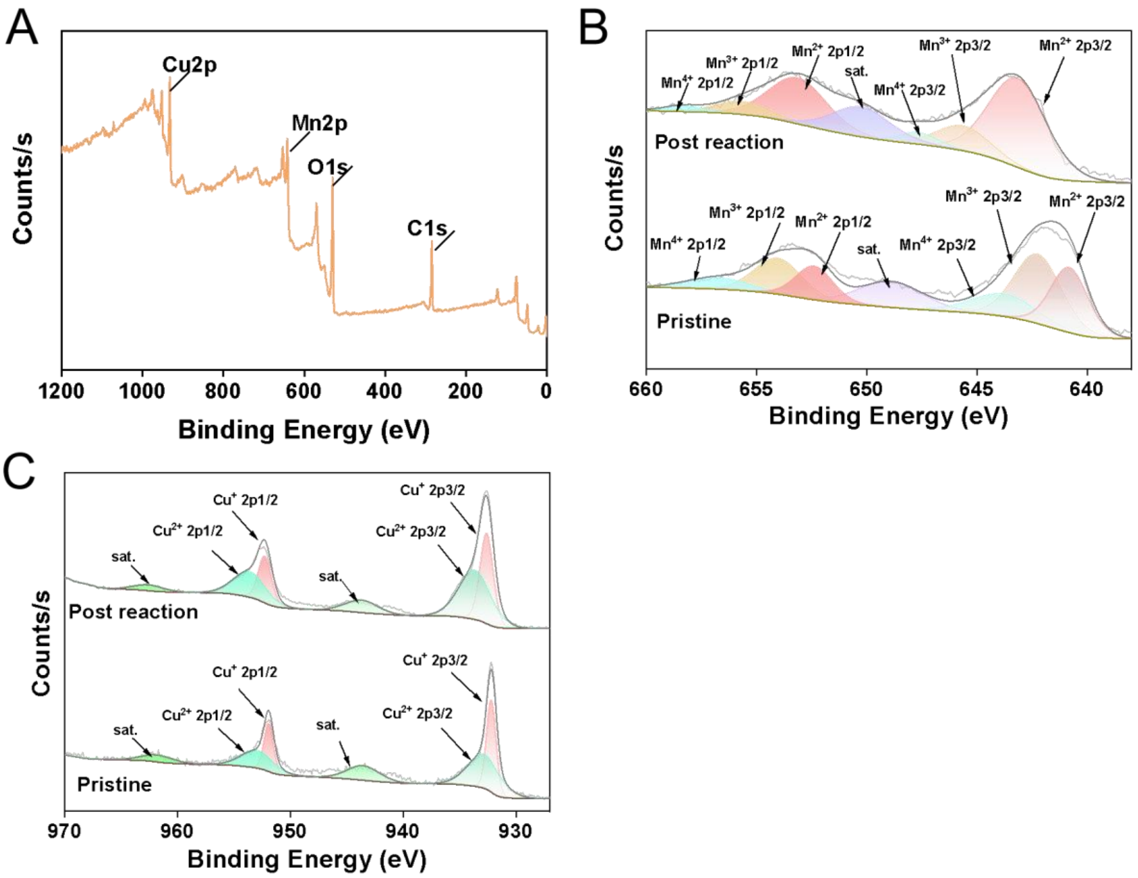

Figure S10. (A) Survey XPS spectrum of CMO after reaction with GSH. (B,C) High resolution Mn2p (B) and Cu 2p (C) XPS spectra of CMO before and after reaction.

Table S3 The quantitative content of $Mn^{2+}/Mn^{3+}/Mn^{4+}$ and the $O_v/(O_v+O_{lat})$ values of CMO before and after reaction with GSH according to the XPS analysis.

CMO	Mn^{2+}(%)	Mn^{3+}(%)	Mn^{4+}(%)	$O_v/(O_v+O_{lat})$
Post reaction	74.7	18.4	6.9	0.79
Pristine	34.1	46.6	19.3	0.54

A

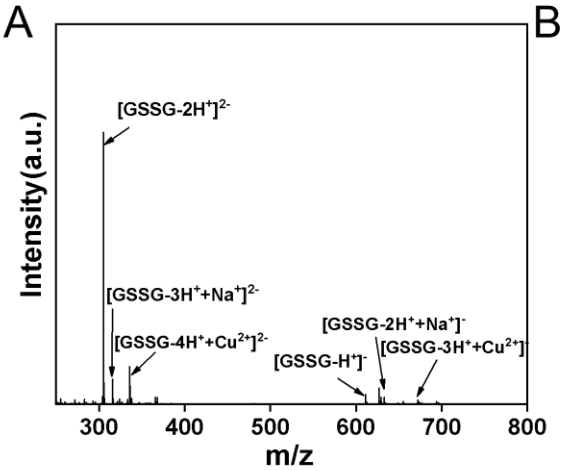

B

Compound	Found	Calculated
$[GSSG-2H^{+}]^{2-}$	305.0686	305.0687
$[GSSG-3H^{+}+Na^{+}]^{2-}$	316.5096	316.5097
$[GSSG-4H^{+}+Cu^{2+}]^{2-}$	335.5253	335.5257
$[GSSG-H^{+}]^{-}$	611.1442	611.1447
$[GSSG-2H^{+}+Na^{+}]^{-}$	633.1262	633.1266
$[GSSG-3H^{+}+Cu^{2+}]^{-}$	672.0583	672.0586

Figure S11. High Resolution Mass Spectrometry (HRMS) analysis of GSH (1 mM) after incubation with CMO (0.1 mg/mL).

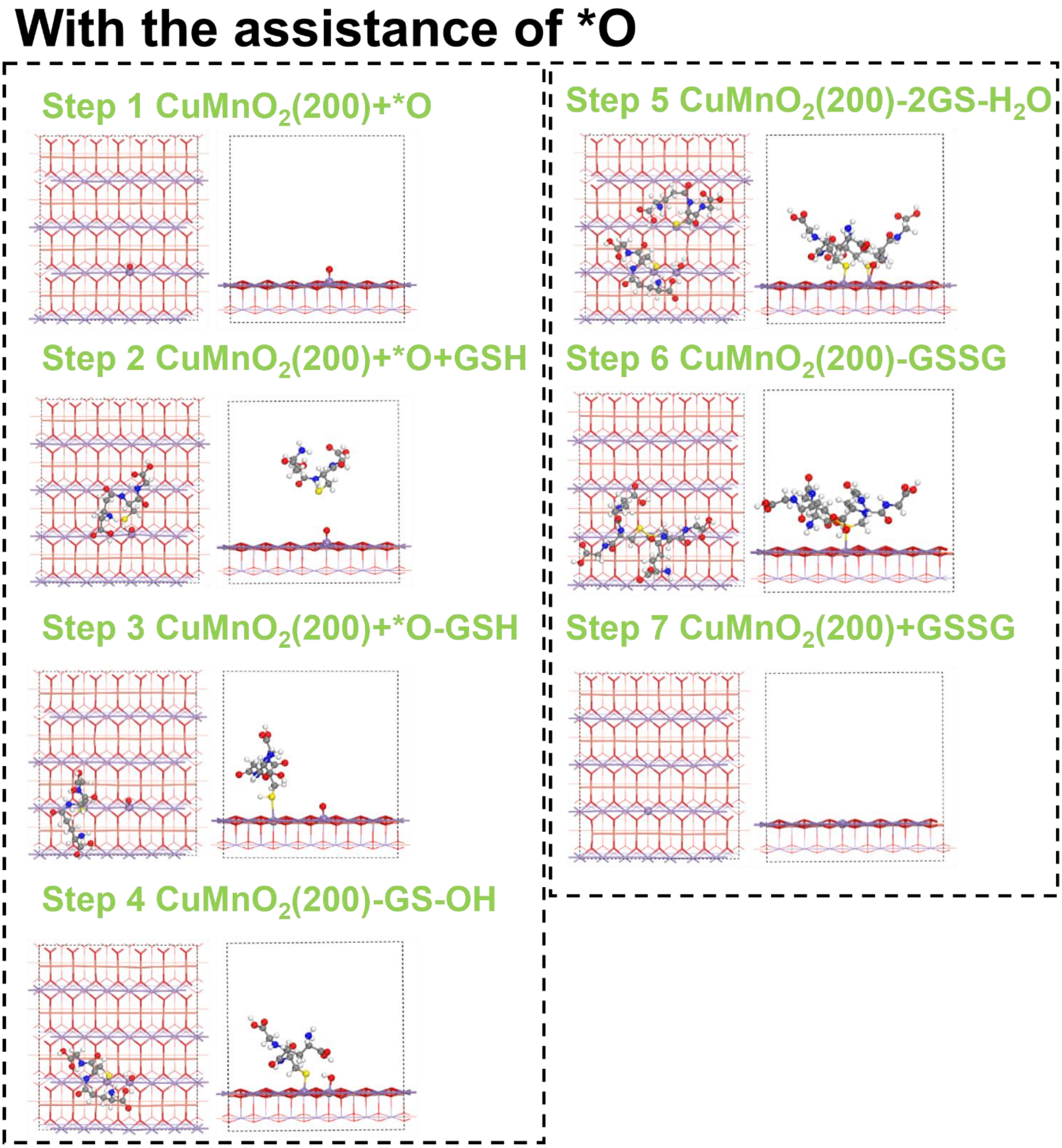

Figure S12. The proposed catalytic mechanism of the oxidation of GSH on the (200) facet of CMO with the assistance of *O. The gray, white, red, blue, yellow, purple, and pink balls represent the C, H, O, N, S, Mn, and Cu atoms, respectively.

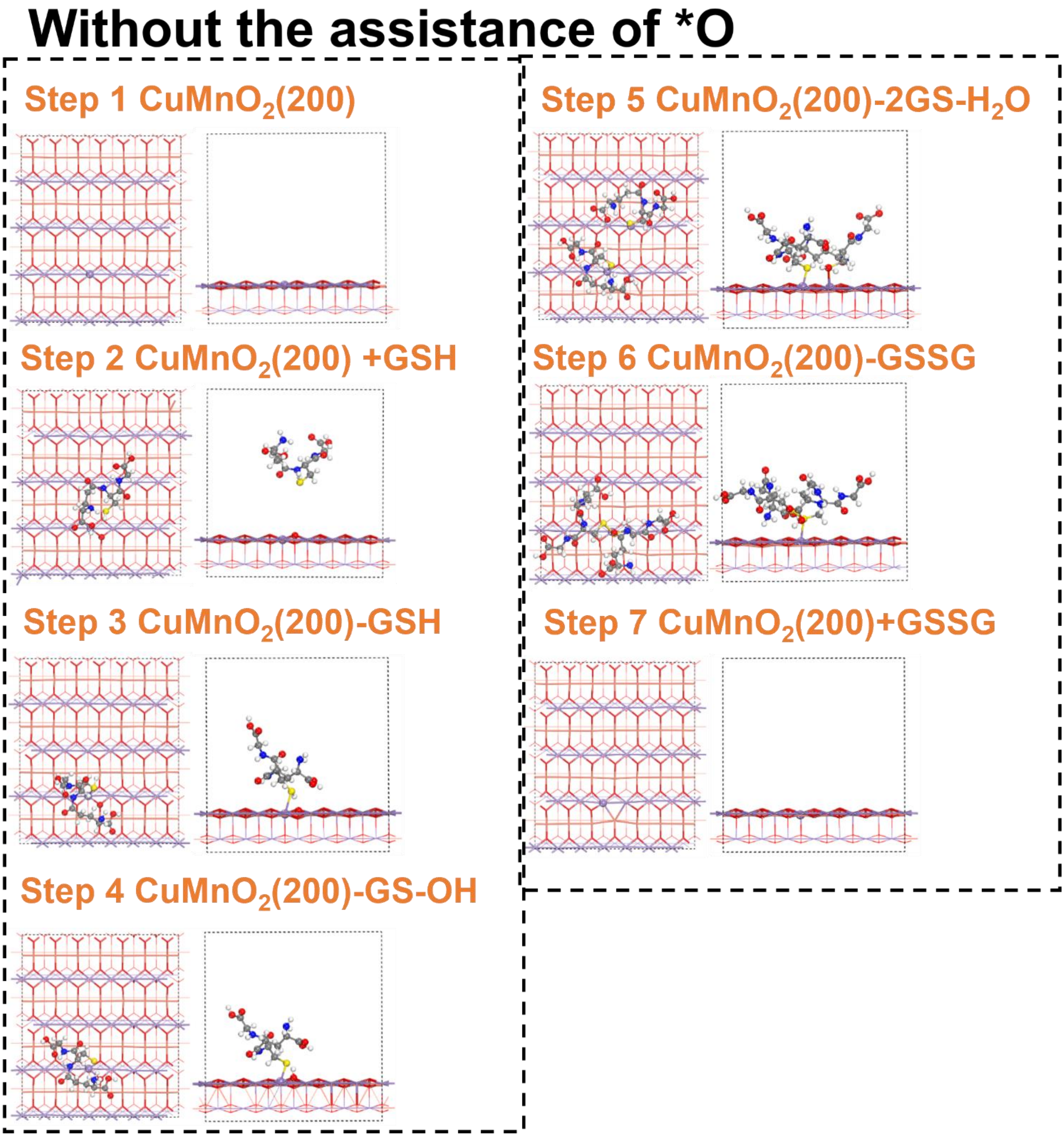

Figure S13. The proposed catalytic mechanism of the oxidation of GSH on the (200) facet of CMO without the assistance of *O. The gray, white, red, blue, yellow, purple, and pink balls represent the C, H, O, N, S, Mn, and Cu atoms, respectively.

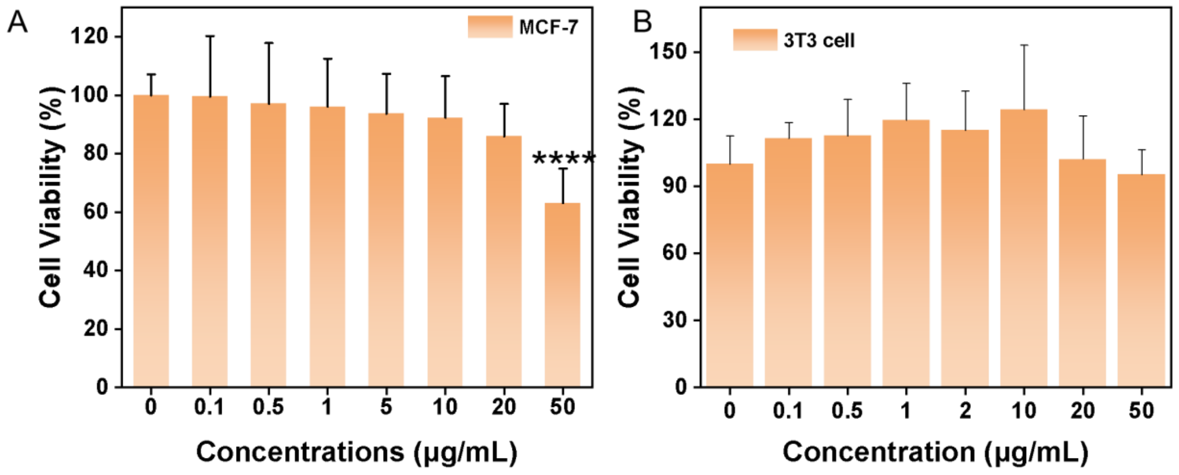

Figure S14. Cell viabilities of MCF-7 cells (A) and 3T3 cells (B) after CMO treatments of different concentrations. All data are presented as means±SD (n = 6 independent experiments). Significance between two groups was assessed by Paired Sample t-test. *P < 0.05; **P < 0.01; ***P < 0.001;****P < 0.0001.

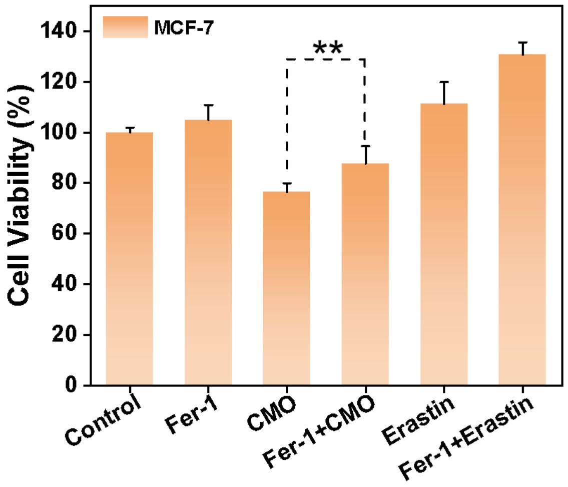

Figure S15. Cell viability of MCF-7 cells after different treatments. Significance between two groups was assessed by Paired Sample t-test. *P < 0.05; **P < 0.01; ***P < 0.001;****P < 0.0001.

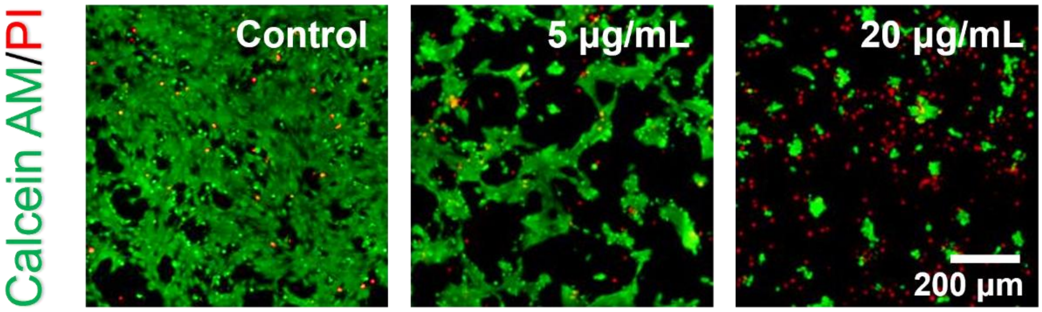

Figure S16. CLSM images of 4T1 cells stained with Calcein AM/PI after incubation of CMO of different concentrations.

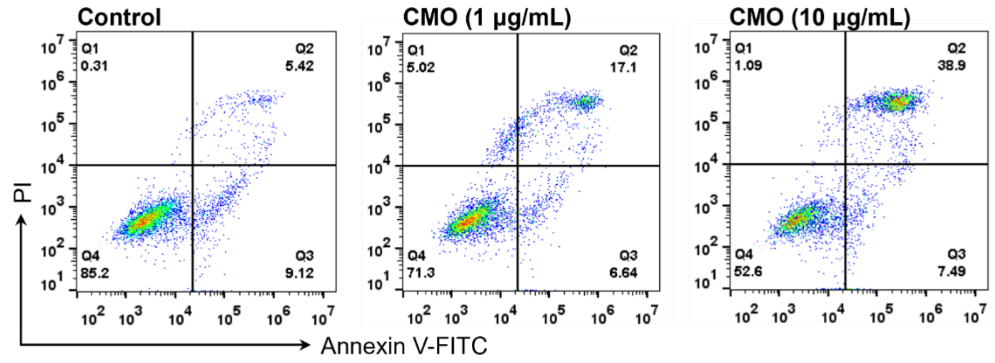

Figure S17. Flow cytometric analysis of Annexin V-FITC/PI stained 4T1 cells after CMO treatments.

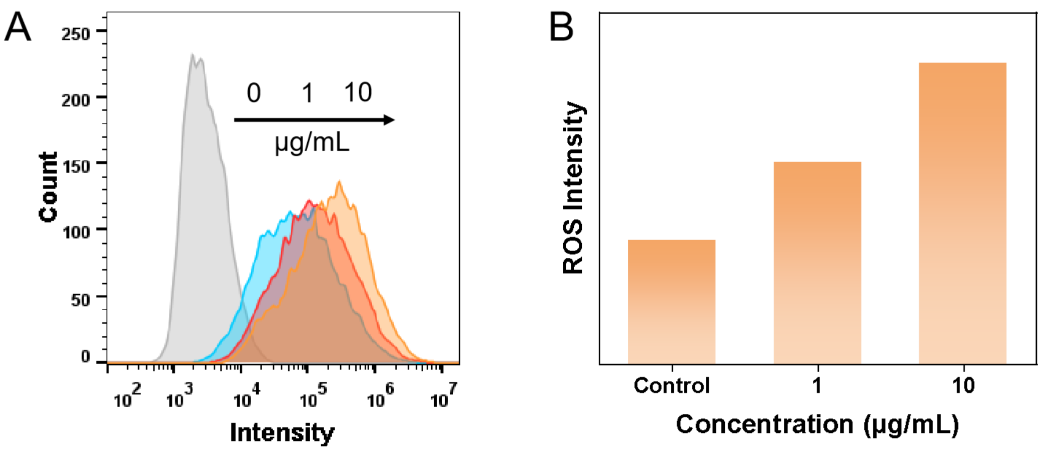

Figure S18. (A) Flow cytometry analysis of DCFH-DA-stained 4T1 cells incubated with CMO of different concentrations. (B) The fluorescence intensity of DCFH in 4T1 cells incubated with CMO.

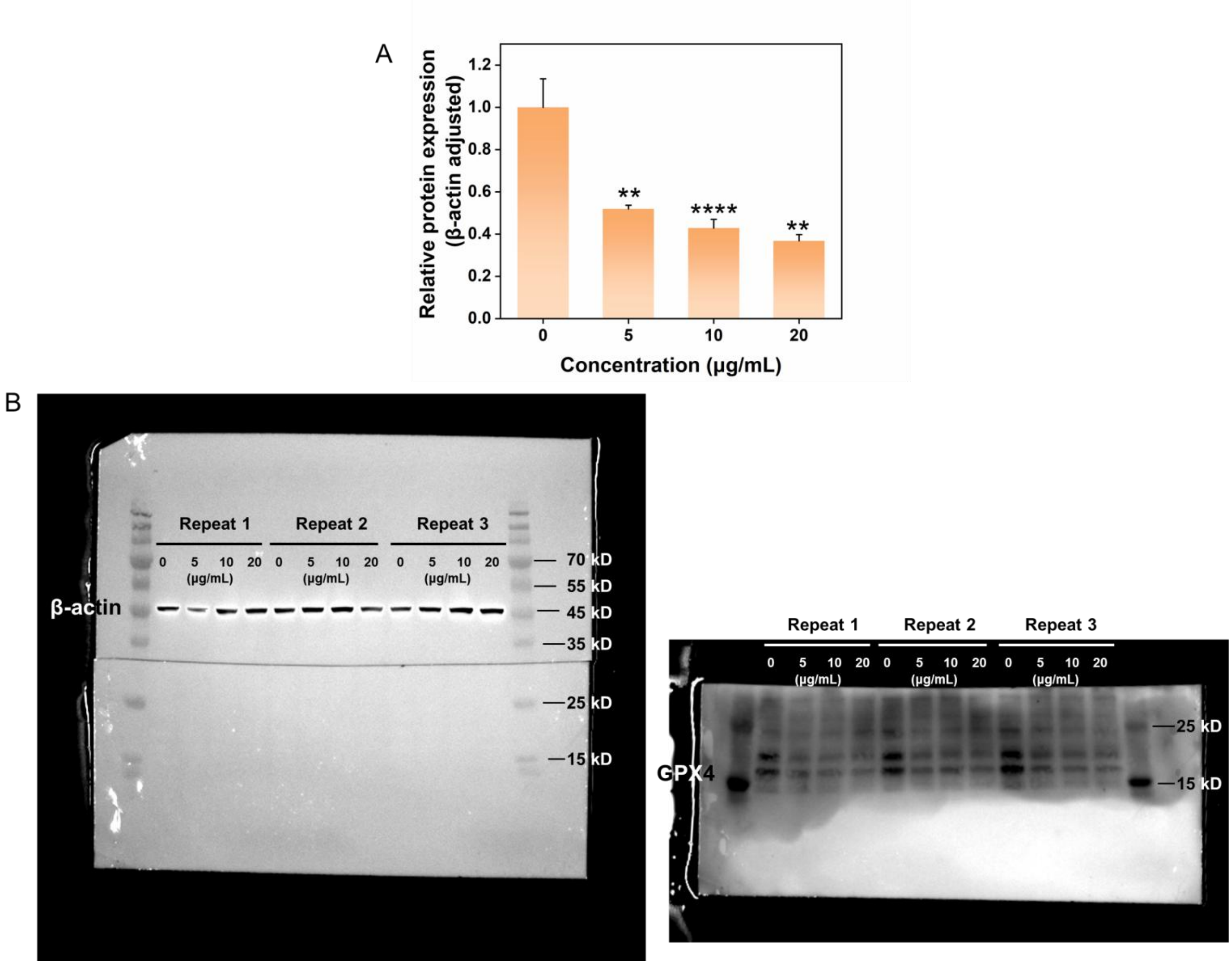

Figure S19. The quantification of Western blot results (A) and the original membranes that were not trimmed in the WB experiment (B). Significance between two groups was assessed by Paired Sample t-test. *P < 0.05; **P < 0.01; ***P < 0.001;****P < 0.0001.

.

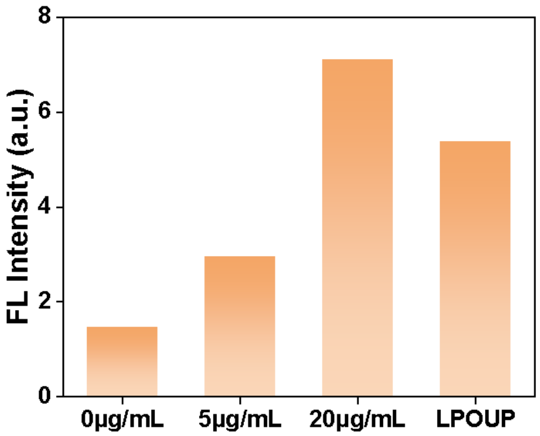

Figure S20. LPO level of 4T1 cells incubated with CMO characterized by the ratio of Q2/Q1.

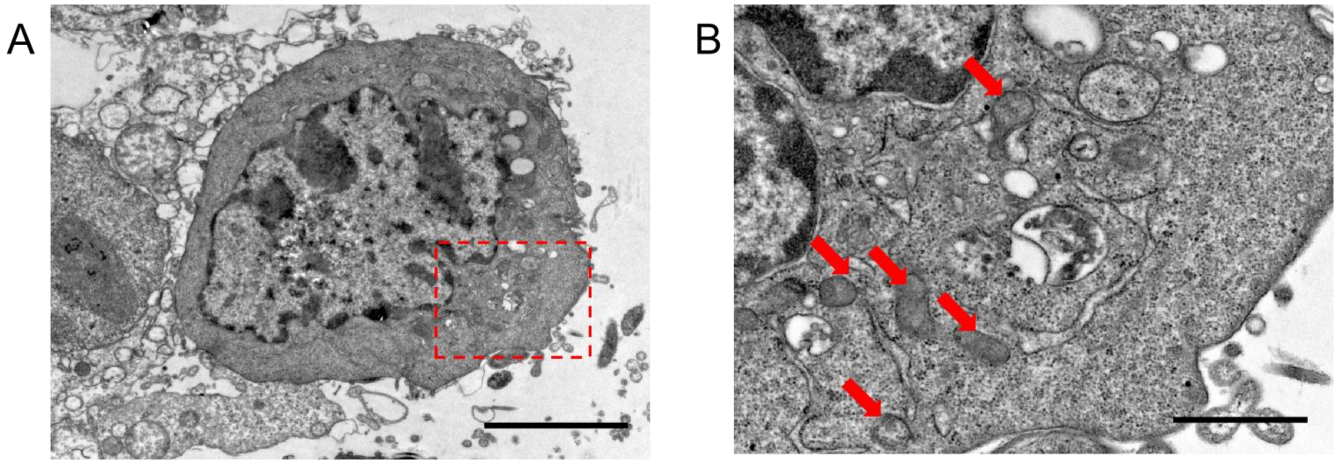

Figure S21. (A) TEM images of intact 4T1 cells. Scale bar = 4 μm. (B) Enlarged image of the red square in (A). Scale bar = 1 μm. (Red arrow: mitochondria)

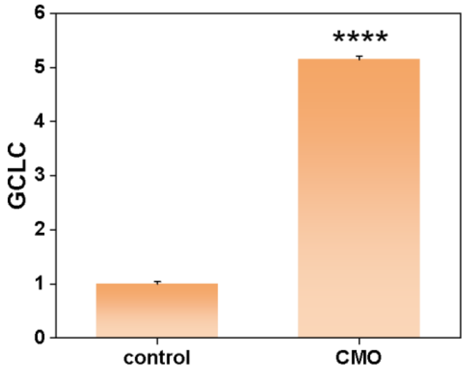

Figure S22. Relative expression of GCLC. Significance between two groups was assessed by Paired Sample t-test. *P < 0.05; **P < 0.01; ***P < 0.001;****P < 0.0001.

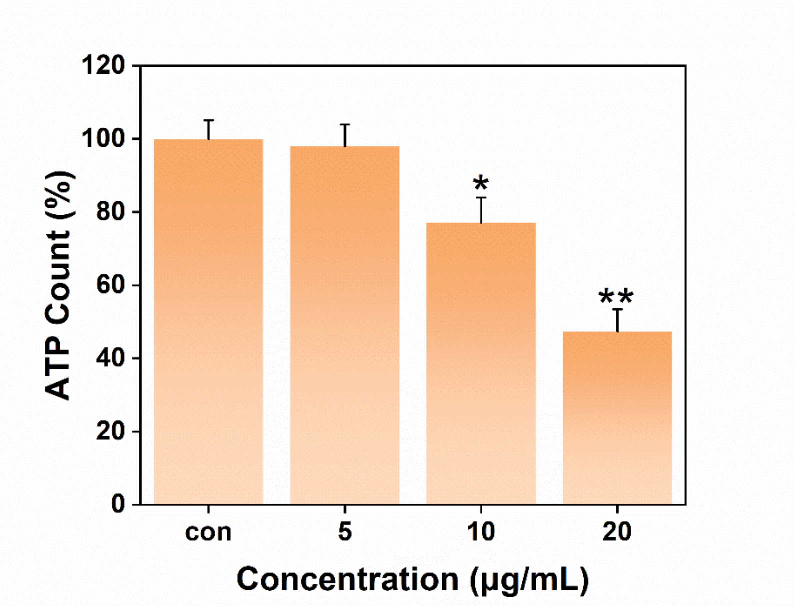

Figure S23. Detection of ATP content of 4T1 cells treated with different concentrations of CMO. Significance between two groups was assessed by Paired Sample t-test. *P < 0.05; **P < 0.01; ***P < 0.001;****P < 0.0001.

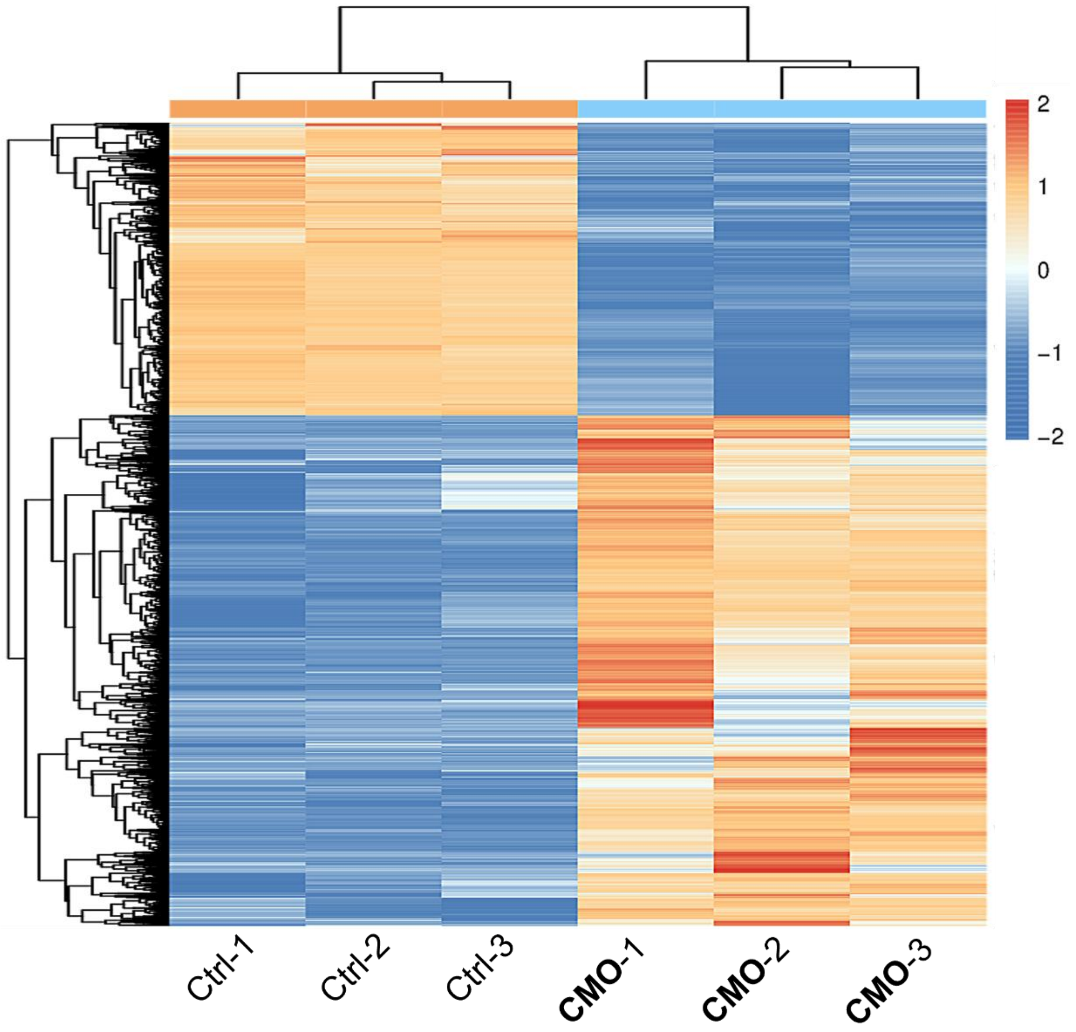

Figure S24. Heatmap showing the DEGs between CMO (10 μg/mL) treated cells compared to the untreated group.

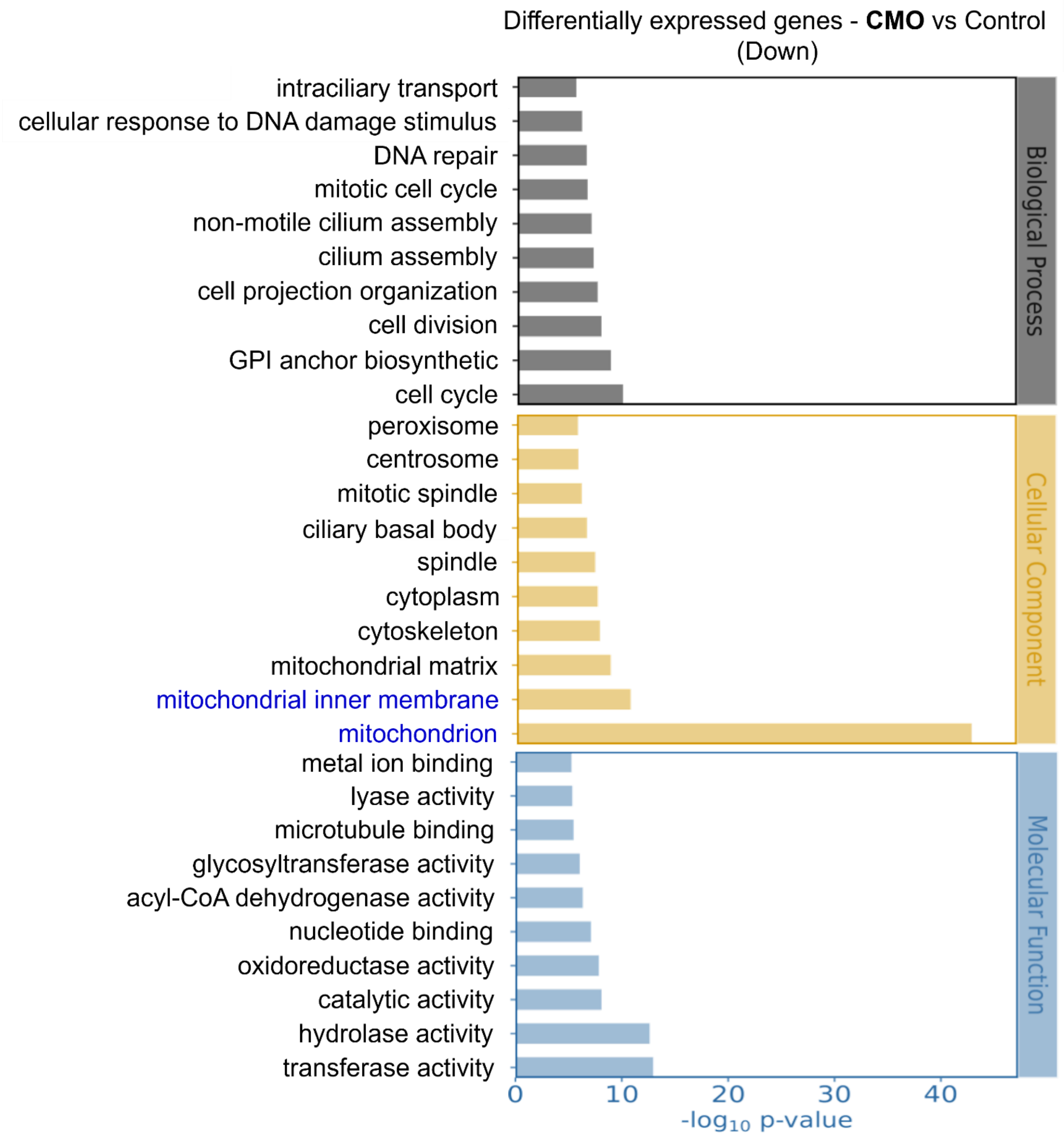

Figure S25. Gene ontology annotations showing the DEGs in 4T1 cells treated with CMO (10 μg/mL) for 18 h (downregulated)..

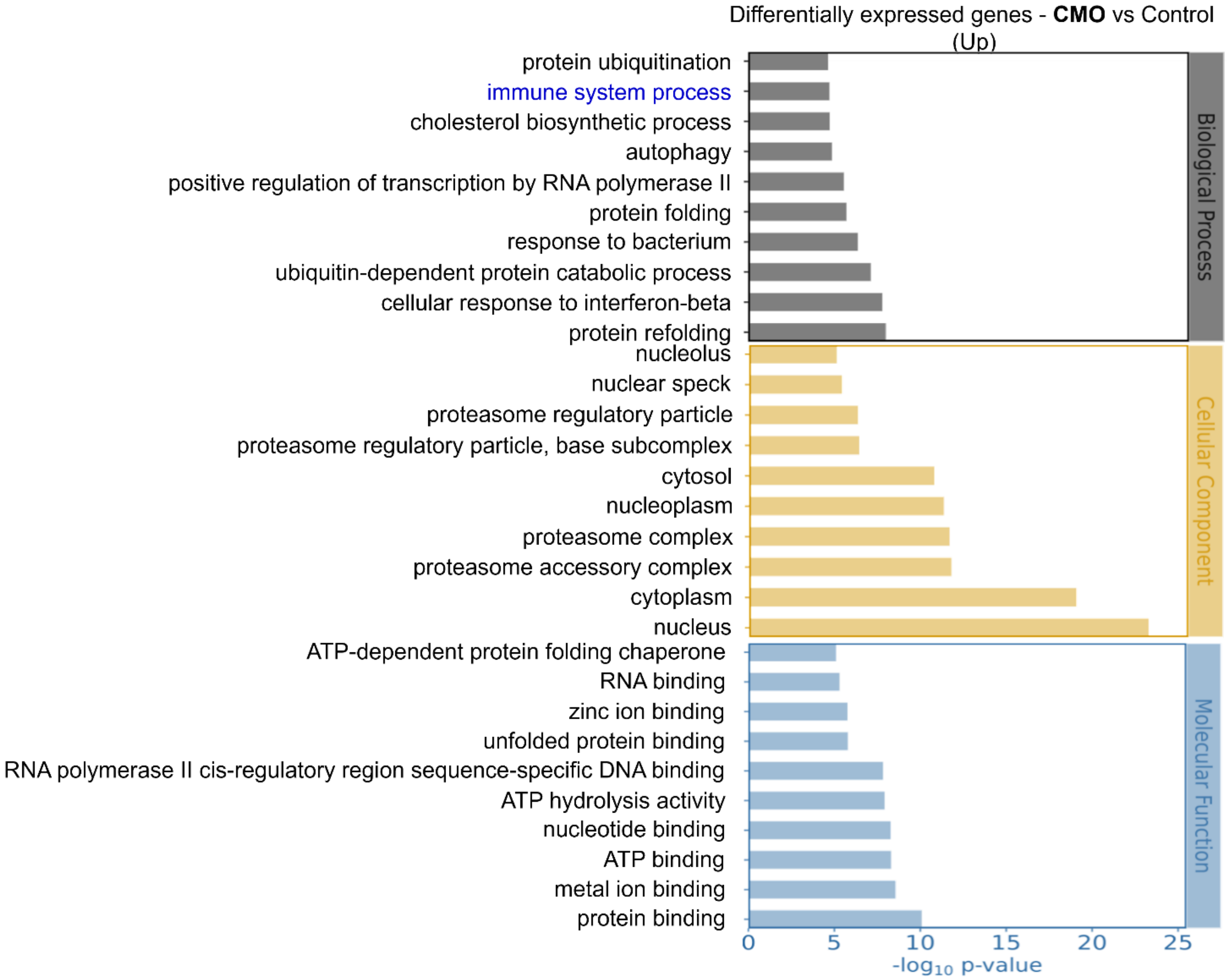

Figure S26. Gene ontology annotations showing the DEGs in 4T1 cells treated with CMO (10 µg/mL) for 18 h (upregulated).

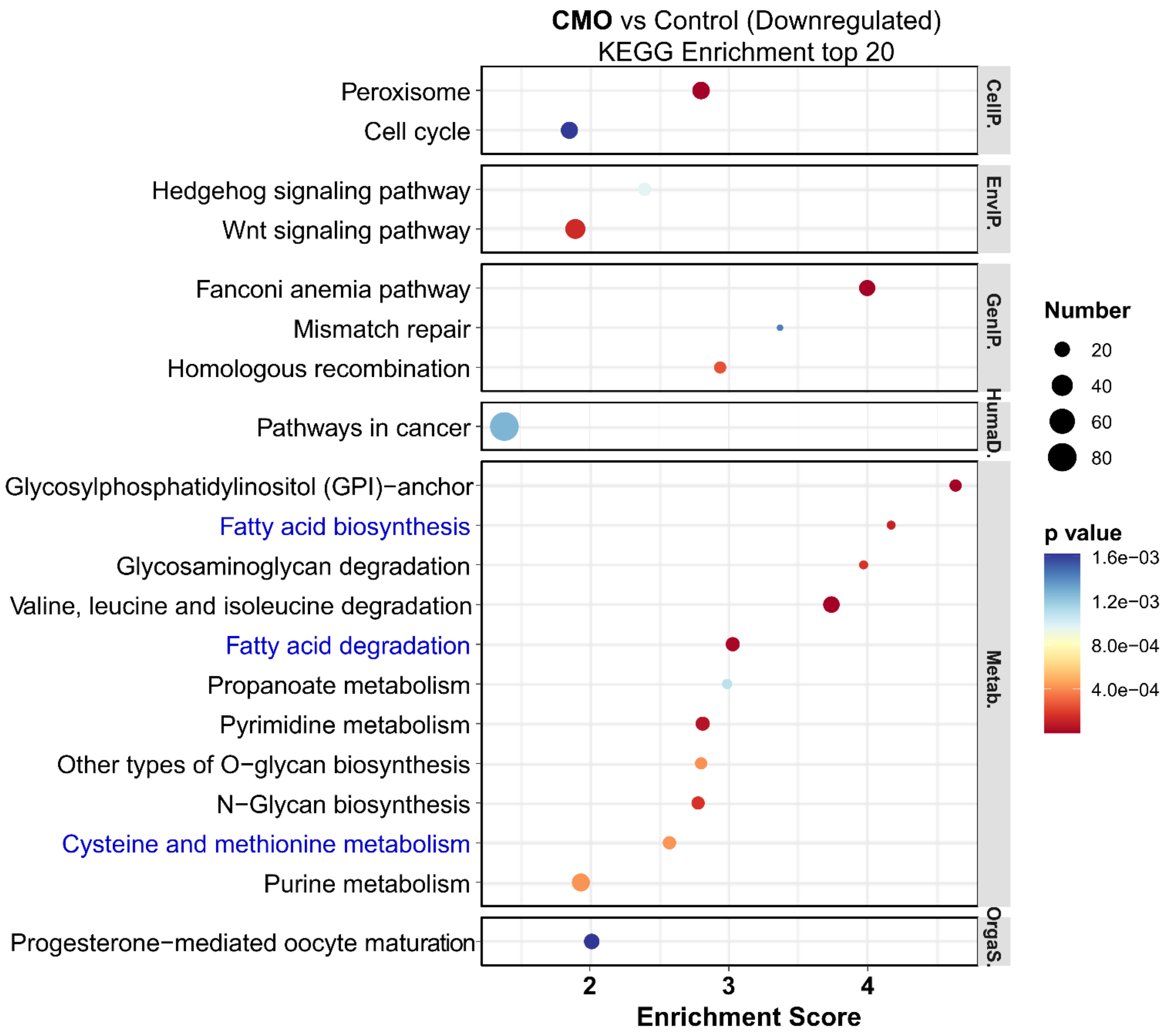

Figure S27. KEGG enrichment analysis (top 20 in total) from CMO group as compared with the control group.

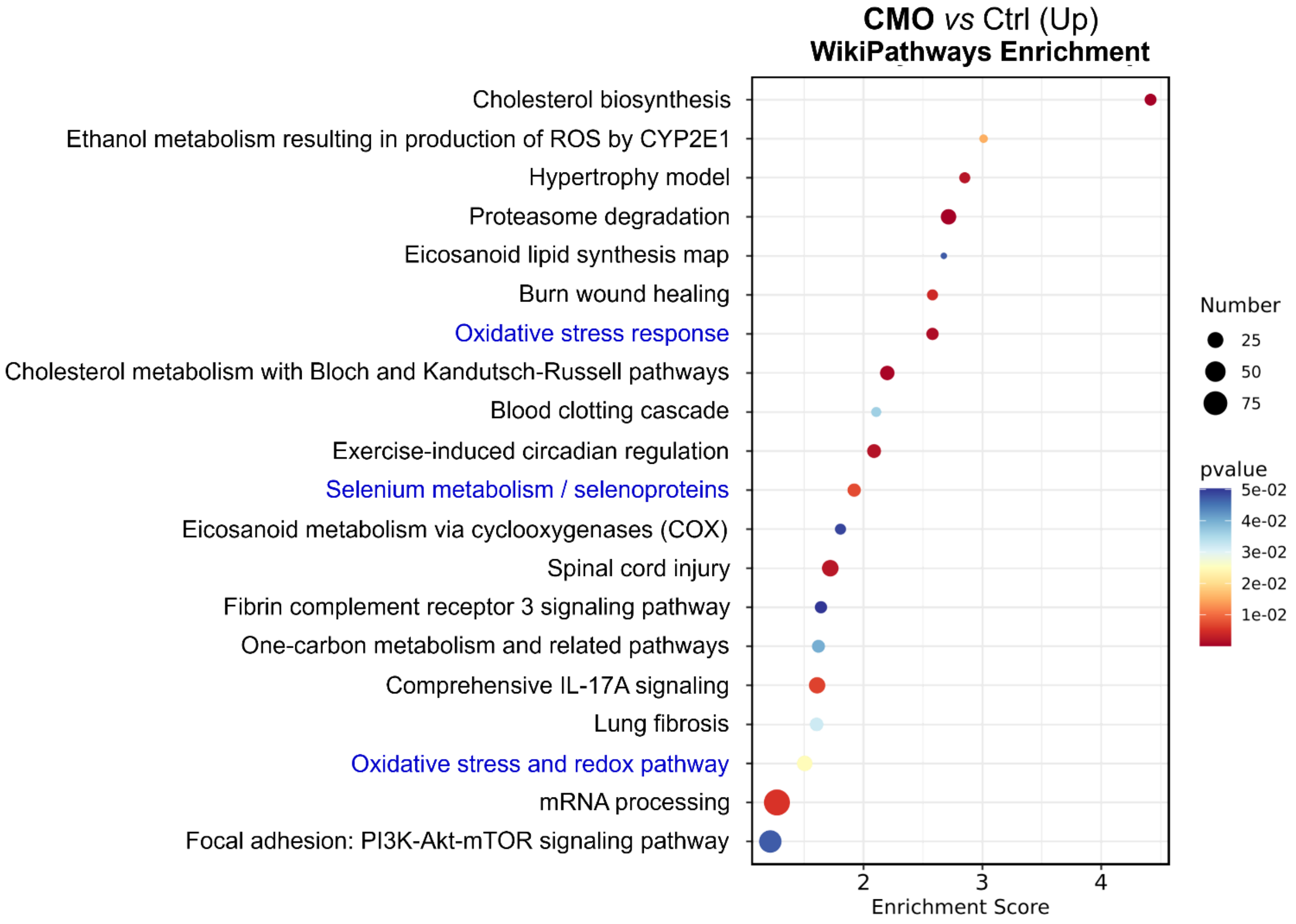

Figure S28. WikiPathways annotations showing the DEGs in 4T1 cells treated with CMO (10 µg/mL) for 18 h.

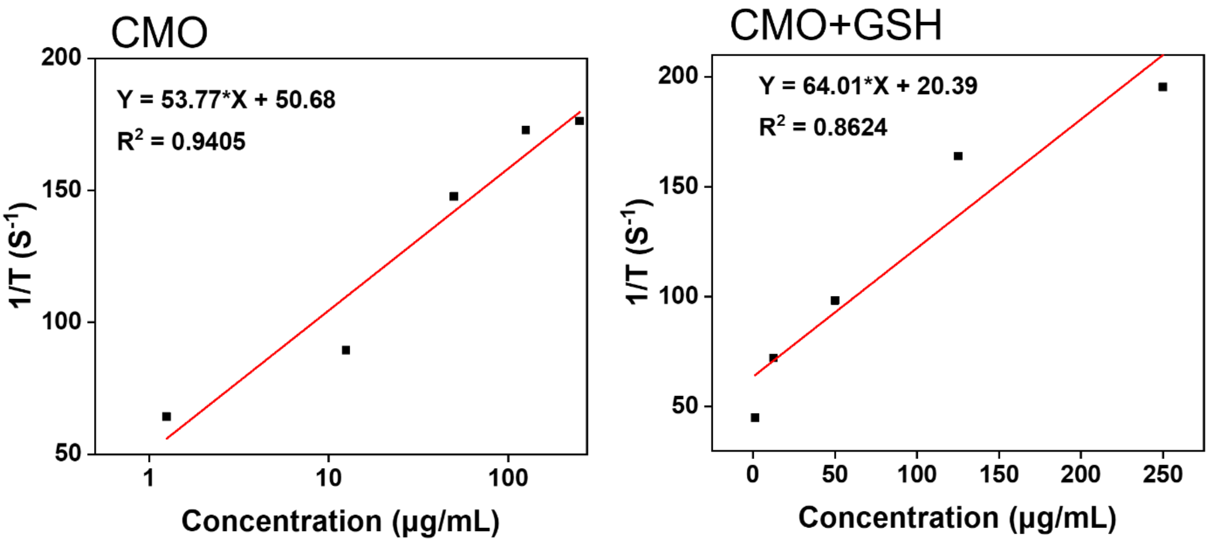

Figure S29. T_1 relaxation rate associated with CMO concentrations with or without the presence of GSH.

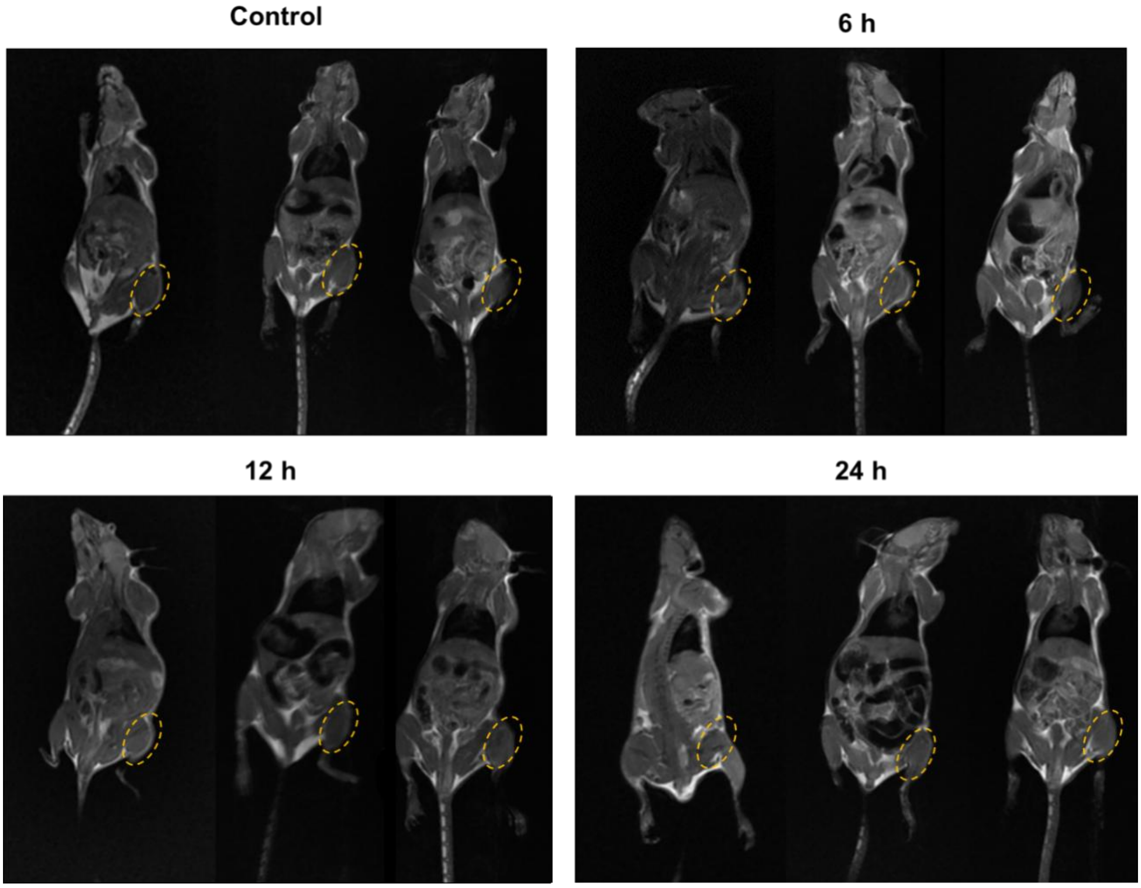

Figure S30. In vivo T_1-weighted MR images of tumor-bearing mice after i.v. injection of CMO (n=3).

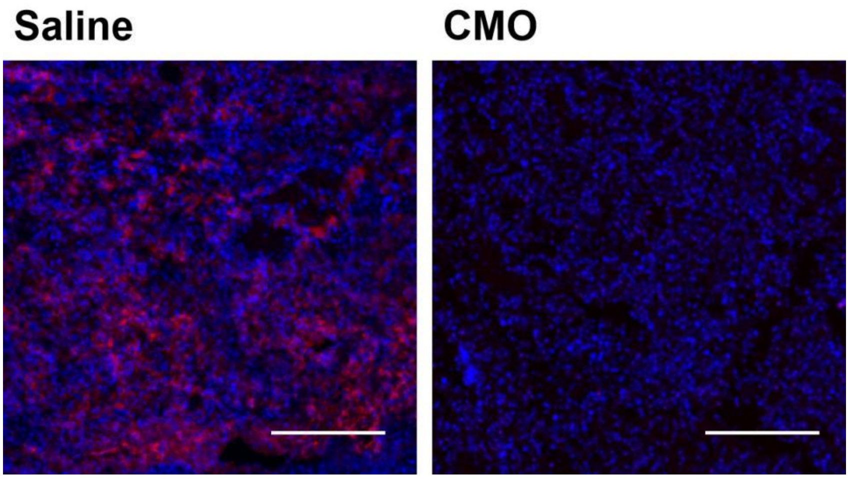

Figure S31. Tumor sections with Ki67 staining after saline or CMO treatment. (Scale bar: 200 μm)

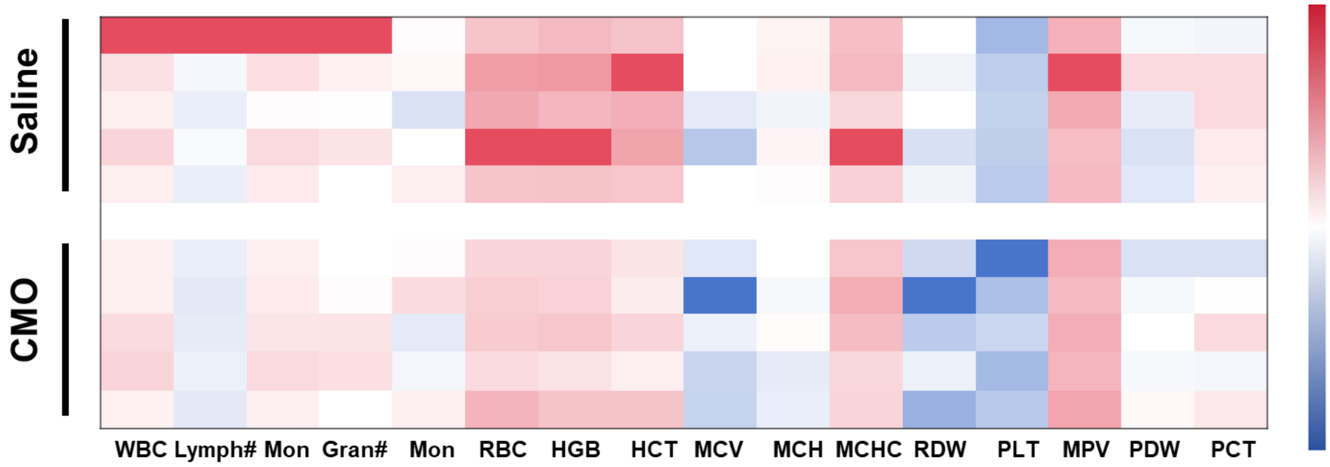

Figure S32. Routine blood results of mice after 14 days of i.v. injection of CMO or saline.

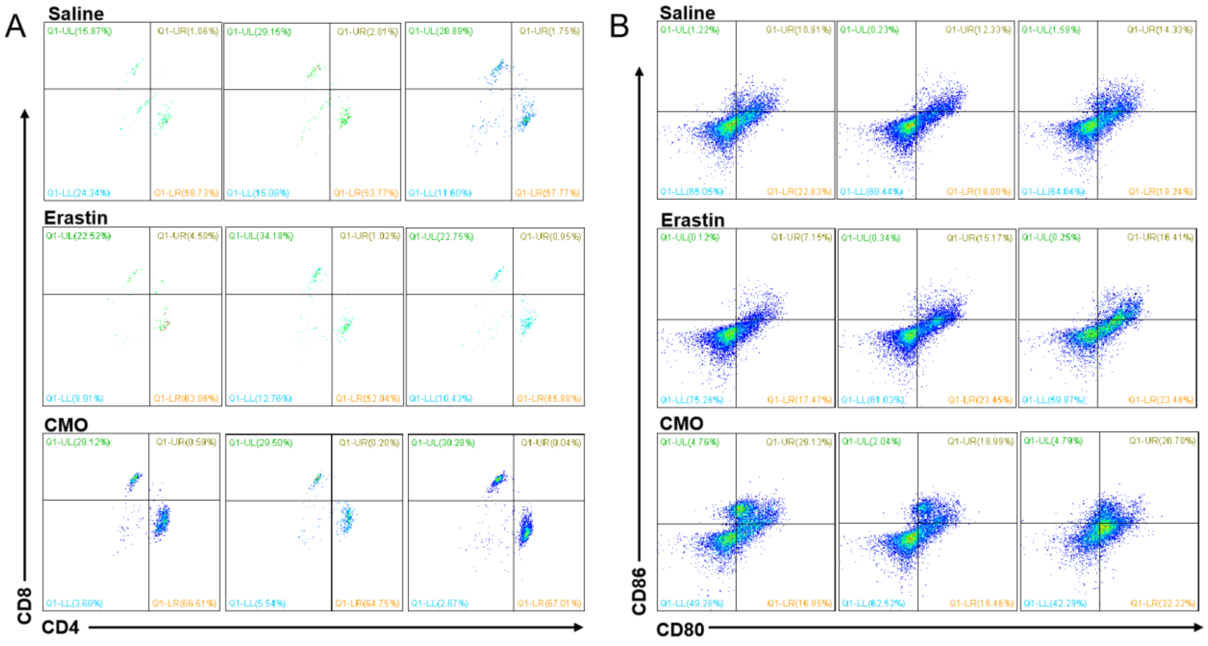

Figure S33. (A) Proportion of $CD4^{+}$ and $CD8^{+}$ T cells in $CD3^{+}$ T cells. (B)The expression of CD80 and CD86 in tumors after CMO and saline treatments.

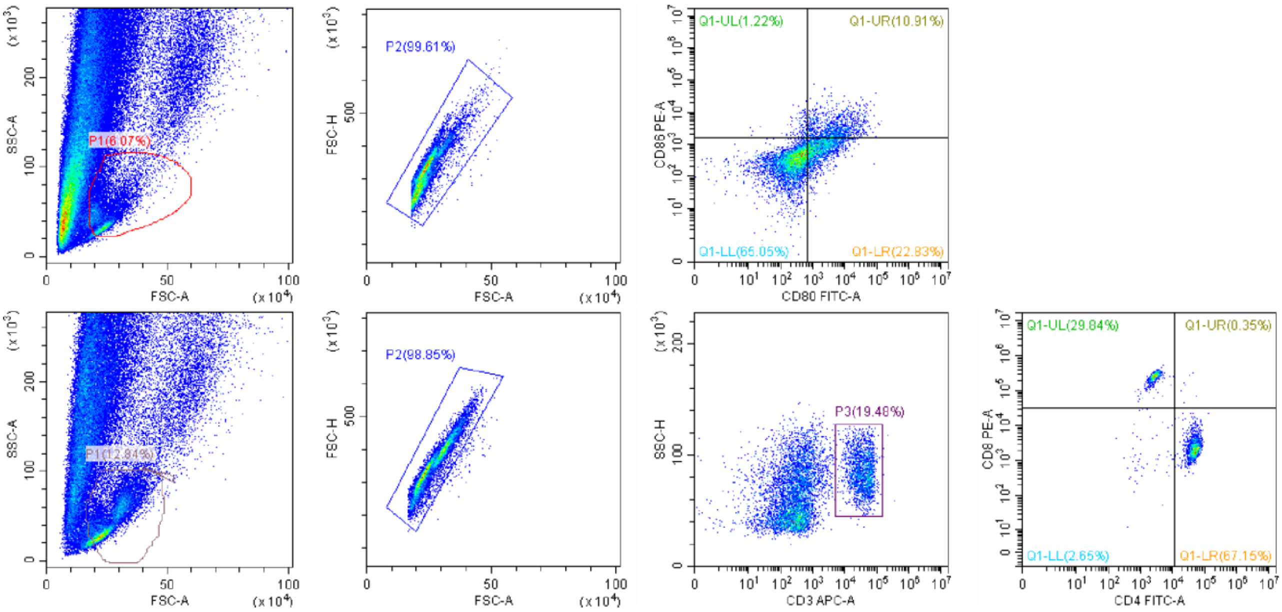

Figure S34. Gating strategy of Figure 9d and Figure S32.

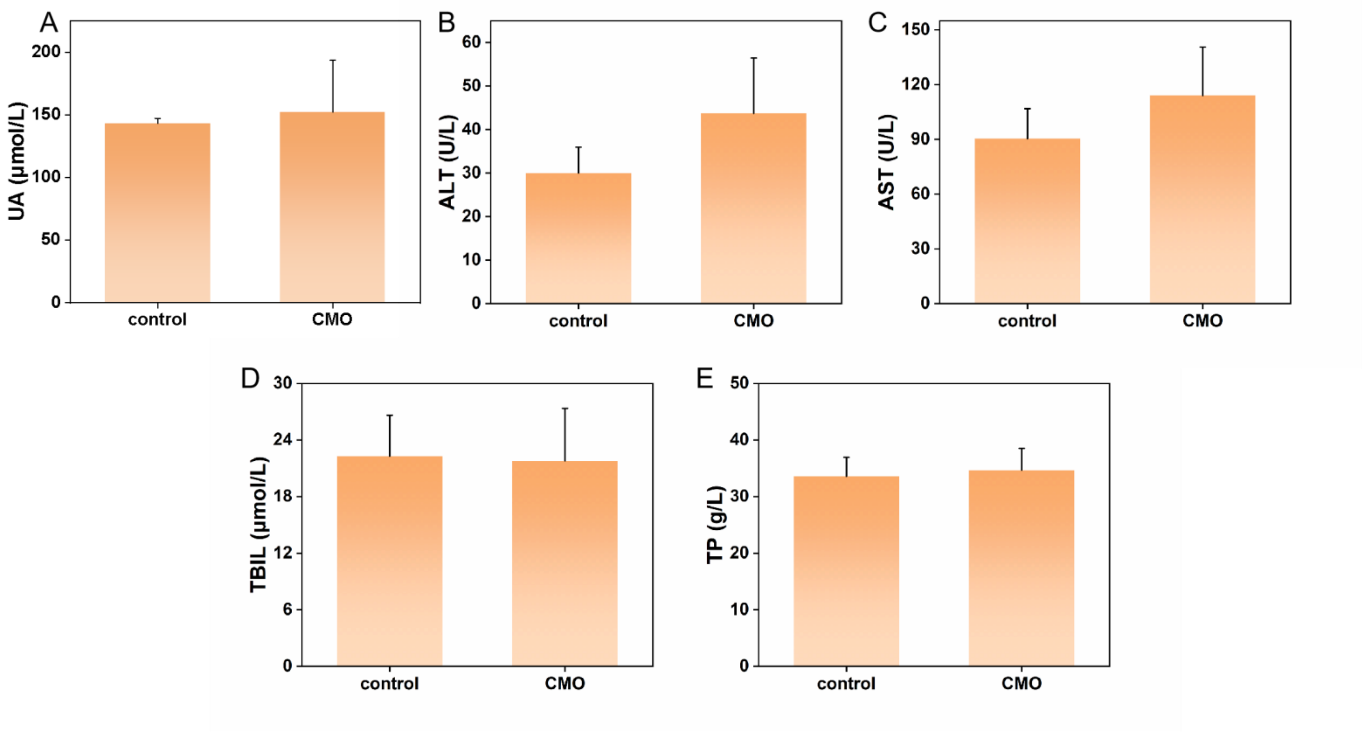

Figure S35. Blood analysis of kidney (A) and liver function (B-E) in different groups of normal mice after 28 days of treatment. (mean ± SD, n = 5)

References

S1 VandeVondele J, Krack M, Mohamed F, Parrinello M, Chassaing T, Hutter J, Quickstep: Fast and accurate density functional calculations using a mixed Gaussian and plane waves approach. Comput Phys Commun. 2005; 167: S103-28.

S2 Goedecker S, Teter M, Hutter J, Separable dual-space Gaussian pseudopotentials. Phys Rev B. 1996; 54: S1703-10. .

S3 Hartwigsen C, Goedecker S, Hutter J, Relativistic separable dual-space Gaussian pseudopotentials from H to Rn. Phys Rev B. 1998; 58: S3641-62.

S4 Krack M, Parrinello M, All-electron ab-initio molecular dynamics. Phys Chem Chem Phys. 2000; 2: S2105-12.

S5 VandeVondele J, Hutter J, Gaussian basis sets for accurate calculations on molecular systems in gas and condensed phases. J Chem Phys. 2007; 127: S114105.

S6 Perdew J. P, Burke K, Ernzerhof M, Generalized Gradient Approximation Made Simple. Phys Rev Lett. 1996; 77: S3865.

S7 Grimme S, Antony J, Ehrlich S, Krieg H, A consistent and accurate ab initio parametrization of density functional dispersion correction (DFT-D) for the 94 elements H-Pu. J Chem Phys. 2010; 132: S154104.
